# Artificial Intelligence-Driven Transformation of Pediatric Diabetes Care: A Systematic Review and Epistemic Meta-Analysis of Diagnostic, Therapeutic, and Self-Management Applications

**DOI:** 10.3390/ijms27020802

**Published:** 2026-01-13

**Authors:** Estefania Valdespino-Saldaña, Nelly F. Altamirano-Bustamante, Raúl Calzada-León, Cristina Revilla-Monsalve, Myriam M. Altamirano-Bustamante

**Affiliations:** 1 Metabolic Diseases Research Unit, National Medical Center “Siglo XXI”, Mexican Social Security Institute (IMSS), Av. Cuauhtémoc, 330, Col. Doctores, Mexico City 06720, Mexico; estefania.valdespinos99@gmail.com (E.V.-S.); cristina_revilla@hotmail.com (C.R.-M.); 2 Endocrinology Department, National Institute of Pediatrics (INP), Av. Insurgentes Sur 3700, Mexico City 04530, Mexico; glutation2020@gmail.com (N.F.A.-B.); servendocrinologia@pediatria.gob.mx (R.C.-L.)

**Keywords:** artificial intelligence, pediatric diabetes, machine learning, continuous glucose monitoring, closed-loop insulin delivery, digital health

## Abstract

The limitations of conventional diabetes management are increasingly evident. As a result, both type 1 and 2 diabetes in pediatric populations have become major global health concerns. As new technologies emerge, particularly artificial intelligence (AI), they offer new opportunities to improve diagnostic accuracy, treatment outcomes, and patient self-management. A PRISMA-based systematic review was conducted using PubMed, Web of Science, and BIREME. The research covered studies published up to February 2025, where twenty-two studies met the inclusion criteria. These studies examined machine learning algorithms, continuous glucose monitoring (CGM), closed-loop insulin delivery systems, telemedicine platforms, and digital educational interventions. AI-driven interventions were consistently associated with reductions in HbA1c and extended time in range. Furthermore, they reported earlier detection of complications, personalized insulin dosing, and greater patient autonomy. Predictive models, including digital twins and self-learning neural networks, significantly improved diagnostic accuracy and early risk stratification. Digital health platforms enhanced treatment adherence. Nonetheless, the barriers included unequal access to technology and limited long-term clinical validation. Artificial intelligence is progressively reshaping pediatric diabetes care toward a predictive, preventive, personalized, and participatory paradigm. Broader implementation will require rigorous multiethnic validation and robust ethical frameworks to ensure equitable deployment.

## 1. Introduction

Type 1 and type 2 diabetes mellitus represent two of the most prevalent chronic conditions affecting children and adolescents. Their rising incidence contributes substantially to morbidity and mortality, adversely influencing growth, quality of life, and long-term prognosis [1,2,3,4]. The reported incidence of type 1 diabetes ranges from 2.5 to 6 per 100,000 children aged 6 to 11 years, with an early peak between 4 and 6 years and a secondary peak between 9 and 12 years [5].

Type 1 diabetes is associated with a broad spectrum of comorbidities that profoundly affect patients’ quality of life, including retinopathy (≈70%), neuropathy (25–33%), nephropathy (15–25%), advanced chronic kidney disease (20–40%), cardiovascular disease (12–14%), and severe hypoglycemia (26%) [6].

Historical evidence from the Diabetes Control and Complications Trial (DCCT/EDIC) demonstrated that intensive glycemic control—maintaining HbA1c below 7.5% over the long term—can reduce major cardiovascular events by approximately 30%, events associated with a mortality risk 4–10 times higher than that of the general population [7]. The incidence of microvascular complications such as retinopathy, neuropathy, and nephropathy decreased by 35–76%, albeit with a concomitant rise in severe hypoglycemic episodes. Notably, 95% of these episodes occurred prior to the introduction of modern insulin analogs, which have since redefined contemporary diabetes therapy.

Approximately 20% of patients with type 1 diabetes develop chronic kidney failure and exhibit a 50% greater likelihood of undergoing lower-limb amputation secondary to diabetic neuropathy [6]. Conversely, type 2 diabetes is linked to a 2–3-fold higher mortality from severe cardiovascular disease compared with the general population, a risk that doubles in the presence of comorbidities such as hypertension, dyslipidemia, or obesity, particularly among patients with suboptimal metabolic control [7].

Beyond its clinical repercussions, diabetes imposes a substantial economic burden on patients, families, and healthcare systems, constituting both a national and global public health challenge. In Mexico, the Mexican Institute of Social Security (IMSS) reported that in 2022, expenditures for diabetes care reached 50.6 billion pesos, with projections exceeding 443 billion pesos by 2030—an alarming 775% increase within less than a decade [8]. Globally, the American Diabetes Association estimated that the total annual cost of diabetes care in 2023 reached $412.9 billion, encompassing hospitalizations and the management of comorbidities. Microvascular complications substantially inflate healthcare expenditures, increasing treatment costs by up to 50%, while lower-limb amputations secondary to diabetic neuropathy may cost as much as $90,000 per patient [9].

This economic burden underscores the urgent necessity of developing innovative strategies that optimize prevention and treatment, aiming to achieve superior metabolic control. Artificial intelligence (AI) has already demonstrated promise in enhancing glycemic regulation, predicting long-term complications years in advance, and promoting a novel medical paradigm grounded in continuous patient education—ultimately reducing comorbidities and both direct and indirect healthcare costs.

Optimal care requires early and accurate diagnosis, personalized treatment, and continuous monitoring, tasks that go beyond traditional monitoring methods such as capillary punctures, fixed insulin schedules, and infrequent clinical visits. Artificial intelligence (AI) offers a data-driven alternative. Machine-learning models fed with clinical, physiological, metabolomic, and behavioral data can forecast hypoglycemia [4], automate insulin titration [10], and predict chronic complications up to a decade in advance [11]. Deep-learning algorithms even exploit non-invasive signals such as QTc and heart-rate variability to detect impending hypoglycemia with high sensitivity [3,4], predict glucose 30 min ahead via regression models trained on real-world data, and boost adherence through interactive digital platforms [1].

These innovations underpin continuous-glucose monitoring, closed-loop “artificial pancreas” systems, telemedicine, and educational apps, interventions shown to improve glycemic control, satisfaction, self-management, and safety [2,12,13]. Nevertheless, most studies are short, single-center, and rarely address equity, long-term validation, or data-governance challenges [14,15,16]. This analysis defines specific areas of opportunity for AI and digital health interventions with great potential benefits in early detection, timely treatment, and especially in reducing long-term occurrence.

**Figure 1:**
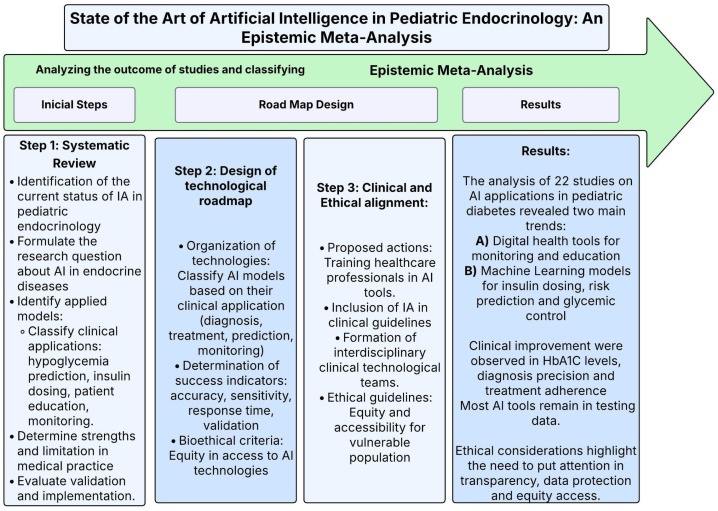
Methodological diagram—state of the art of artificial intelligence in pediatric endocrinology: an epistemic meta-analysis. This diagram outlines the methodology used in the meta-analysis of the application of artificial intelligence (AI) in pediatric endocrinology. It achieved this by dividing it into three steps: (1) a systematic review, where it was possible to identify new models that were being evaluated. (2) A design of the methodological path to classify AI models according to their clinical applications and equity criteria. (3) The clinical and ethical alignments in the medicine practice, where the emphasis is placed on training actions in the inclusion of AI in clinical guidelines.

This systematic review therefore synthesizes current evidence on AI and other emerging digital technologies in pediatric diabetes care, comparing their diagnostic accuracy, risk prediction, therapeutic personalization, and clinical effectiveness with traditional methods. We (i) map AI applications across diagnosis, monitoring, treatment, and education; (ii) quantify clinical, behavioral, and educational outcomes; (iii) identify the most robustly validated approaches and their limitations; and (iv) examine ethical and equity implications to guide safe, inclusive implementation. The guiding question for this review: to what extent AI is transforming the diagnosis, treatment, and overall management of diabetes in children and adolescents compared with conventional care, and how these tools influence diagnostic accuracy, therapeutic personalization, and real-world effectiveness of digital therapies (Figure 1).

## 2. Materials and Methods

We performed a PRISMA-guided systematic review to evaluate artificial intelligence (AI) applications in pediatric diabetes care. Searches spanned PubMed, Web of Science, and BIREME from database inception to February 2025. Only English published studies were considered eligible for inclusion.

As previously stated, the study is divided into three main phases, and (Figure 1) demonstrates the structure and central elements of each phase of the paper.

### 2.1. PIO and PRISMA Strategy

A systematic review of existing medical articles was carried out until February 2025, with the implementation of a PIO strategy (Participants, Intervention, and Results) and a PRISMA evaluation (viable elements for systematic review and meta-analysis). The methodology of this article was instrumental in structuring the review and providing an answer to the research question raised about innovation in artificial intelligence and endocrinology: How is artificial intelligence transforming diagnosis, treatment, and management of diabetes mellitus in children and adolescents compared to traditional methods, and what impact does it have on diagnostic accuracy, risk prediction, treatment personalization, and the effectiveness of digital therapies?

**Figure 2:**
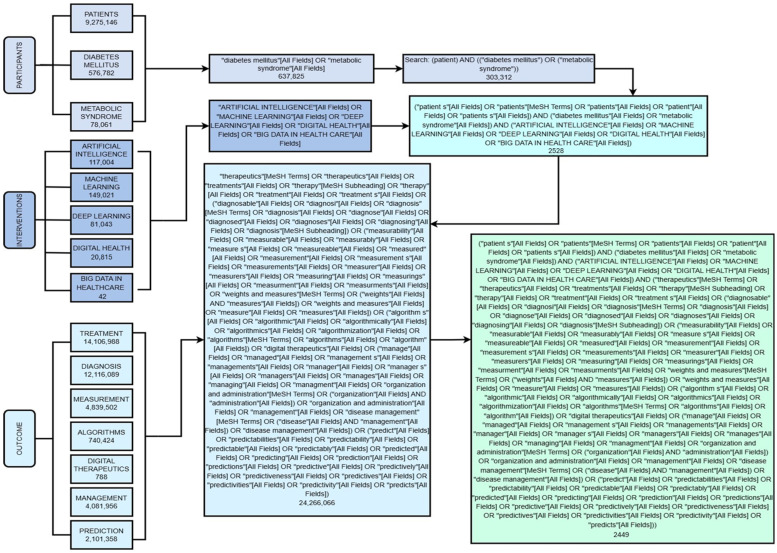
Decision tree of the PIO search strategy. This figure summarizes the application of the PIO strategy (population, intervention, outcome) to structure and integrate search terms into the selected databases. This shows how pediatric population, artificial intelligence-driven interventions and clinical outcomes are systematically combined to answer the research question on the role of AI in managing pediatric diabetes. In doing so, a transparent and reproducible research process is assured.

**Figure 3:**
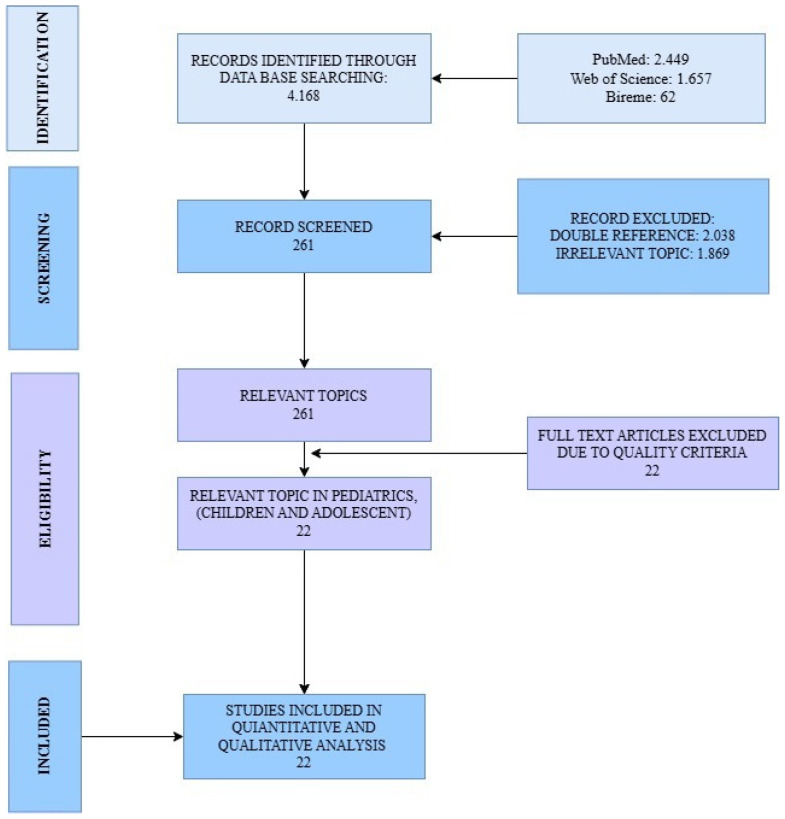
PRISMA flow diagram. The figure illustrates the stepwise selection process of articles, from the initial identification of records across all databases, through screening and eligibility assessment, to the final inclusion of studies in the review.

The methodological structure of this process is represented in (Figure 2 and Figure 3), which serve as a guide for visualizing the objectives, the analysis process, and the route followed to obtain the results, discussion, and conclusions.

### 2.2. PIO Strategy

A PIO strategy was planned for the implementation of this systematic review. The following terms were used to analyze and answer the research question in terms of: Participants, Interventions, and Outcomes. The decision tree diagram to carry out this strategy is shown in (Figure 2) together with the search algorithms with MeSH terms collected during the review. Unlike the traditional PICO model, the “Comparision” element was not developed, as the objective of this review was not to compare AI with other interventions but to explore the transformative impact of AI in the field of pediatric endocrinology. The components of the PIO strategy derived from the central question were as follows: for the P section, population-based studies were included in patients with diagnoses of endocrine diseases with special emphasis on Diabetes, P = patient, diabetes mellitus, metabolic syndrome; for the Intervention, studies were included with reference to new technologies: I = artificial intelligence, machine learning, deep learning, digital health, and big data in healthcare; for the Outcomes, studies were included in relation to the Treatment and diagnosis of endocrinological diseases of interest: O = treatment, diagnosis, measurement, algorithms, digital therapeutics, management, and prediction.

### 2.3. Search Strategy

Search terms included combinations of keywords related to “pediatric,” “children,” “adolescents”, “diabetes mellitus”, “type 1”, “type 2”, “artificial intelligence”, “machine learning”, “deep learning”, and “clinical outcomes”. Boolean operators (AND, OR) and controlled vocabulary (MeSH) were adapted for each database. The full search is illustrated in (Figure 2).

#### Temporal Scope and Historical Context of the Search Strategy

Due to the rapid evolution of artificial intelligence and the emerging digital technologies applied in health, there was no lower time limit established for the search strategy. Older studies were intentionally included in this review to provide historical context for understanding the progressive development of computational, algorithmic, and digital approaches to diabetes management. Although there is methodological diversity in the different publication periods, the incorporation of these studies is relevant to chart a technological trajectory that supports contemporary applications. For this reason, early studies were preserved both in the tables and in the narrative synthesis.

### 2.4. Study Selection

Records were exported to Mendeley; duplicates were discarded automatically and by manual review. Two reviewers screened titles/abstracts, then full texts. Inclusion criteria were: (i) original research; (ii) pediatric population; (iii) AI intervention; and (iv) reported clinical, diagnostic, or behavioral outcomes. Regarding studies exclusively focused on adult populations, they were excluded unless their primary objective addressed methodological frameworks, predictive models, or digital technologies with direct applicability to pediatric diabetes care. All disagreements were resolved by consensus.

#### Population Stratification and Inclusion Criteria

**Figure 4:**
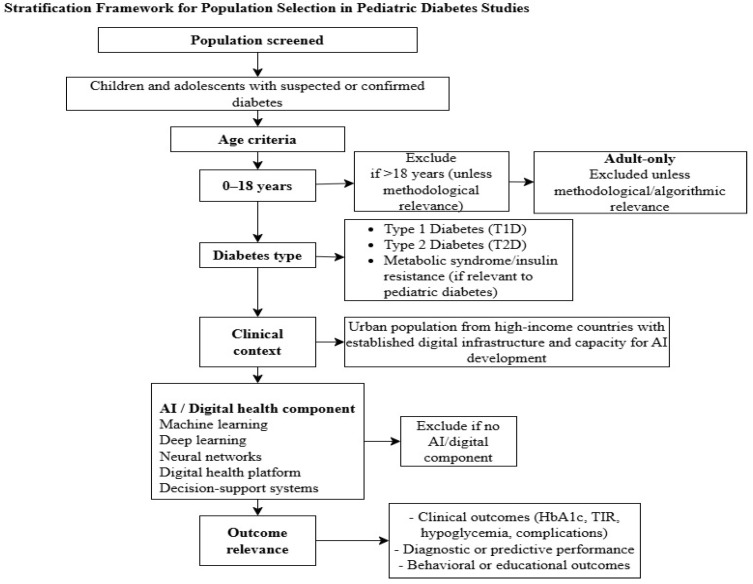
Stratification framework for population selection in pediatric diabetes studies. This figure illustrates the stratification algorithm used to define the study population. This includes criteria for age (0–18 years), type of diabetes (type 1, type 2, or related metabolic conditions), clinical context, and presence of either AI or digital health components. The framework explains why pediatric, mixed-age, and adult-only studies should be excluded or not, ensuring that only populations and methodologies with direct relevance to childhood diabetes care are included in the synthesis.

For this review, the study population was stratified into two categories to ensure conceptual clarity: (i) studies with a strictly pediatric population (0–18 years old) with type 1 and 2 diabetes mellitus; and (ii) mixed studies (adolescents/adults). Studies with a strict pediatric population were included without restriction. They were used to evaluate AI-based health interventions for diabetes diagnosis by monitoring treatment, prediction, or education. Mixed-age studies were included only when they met the following conditions: a) the results of the pediatric population were different from those of the adult population and reported independently; b) the purpose of the study was the development or validation of AI models with a direct methodological application in the care of pediatric diabetes (Figure 4).

### 2.5. Quality Appraisal

Each article was assessed using a 27-item PRISMA-derived checklist (80% required). All 22 studies meeting this threshold were included in the meta-analysis (Figure 3).

### 2.6. Data Extraction and Synthesis

For each study, we captured design, sample size, AI technique, comparator, primary outcomes (e.g., HbA1c change, time-in-range, predictive accuracy), and equity considerations. Heterogeneity in design and outcome measures precluded meta-analysis; findings are therefore summarized narratively and in tabular form.

This streamlined methodology ensured a comprehensive, reproducible assessment of how AI is reshaping pediatric diabetes diagnosis, treatment personalization, and risk prediction relative to conventional care.

### 2.7. Classification of Artificial Intelligence Models Used in the Included Studies

Since the central focus of this review is artificial intelligence, the included studies were analyzed and classified according to the type of AI model used. Thus, it facilitates the interpretation of data and approaches contributing to reported improvements. We identified studies with machine learning models that include supervised algorithms, such as decision trees, vector support machines, and regression models, as well as those focused on deep learning. These latter models were mainly based on artificial neural networks and multilayer neural architectures, which were predominantly used for the prediction of critical hypoglycemia events. Another category was studies that used hybrid models. These types of research integrated physiological data into algorithms supported by AI alongside systems for clinical decisions embedded in digital platforms.

This classification allows a more synthetic understanding of how different approaches to artificial intelligence were applied in an objective way. However, a direct comparison between the performance of each model is not established.

### 2.8. Integral Epistemic Meta-Analysis: Exploring the Application of AI in Management of Pediatric Diabetes

A comprehensive epistemic meta-analysis represents an approach for the analysis and synthesis of existing knowledge derived from heterogeneous studies. Therefore, it allows the assessment of both quantitative and qualitative results as well as theoretical frameworks, methodologies, and analytical structures underpinning each study [17].

Epistemic meta-analysis focuses on the systematic integration of knowledge, methodological approaches, validation strategies, and clinical applicability of AI-driven interventions. This approach is suitable for evolving emerging fields such as AI in pediatric diabetes, where methodological diversity and non-comparable outcomes limit statistical aggregation.

Due to the variability of the interventions, the outcomes, the time variation in the follow-up, and the results, the methodology was not a statistical meta-analysis. A compendium of effects was made with estimations of the effects of AI on HbA1c reduction, improvements in range time, and predictive accuracy. Thus, this review focuses on the directional tendency and not the size of the effect, providing an overall picture.

The application of AI in pediatric endocrinology still presents a very marked methodological diversity, since most studies have a report of results with very small and variable populations that use various applied technologies that make difficult a strict quantitative synthesis.

This differs from a traditional meta-analysis, which requires quantitative data and comparable elements. For this reason, an epistemic integration was developed to establish a coherent framework to identify patterns, strengths, limitations, and gaps in the current evidence.

The purpose of this integrated epistemic meta-analysis is to provide a comprehensive understanding of the current state of knowledge regarding artificial intelligence in pediatric diabetes, highlighting its clinical potential, methodological challenges, and areas of opportunity for future research. Additionally, this approach aims to contribute to the progressive standardization and harmonization of outcomes, thus facilitating more robust quantitative meta-analyses as the field matures.

## 3. Results

### 3.1. Results

#### 3.1.1. State of the Art of Diabetes and Artificial Intelligence

A total of 4168 records were identified through the selected databases following the predefined PIO strategy (Figure 2) and PRISMA guidelines (Figure 3). Among these, 2038 duplicates were removed, and an additional 1809 records were excluded after title and abstract screening for irrelevance to the research question. A standardized PRISMA-based quality appraisal was performed using the following weighted criteria: (a) clarity of objectives, (b) congruence between the research question and objectives, (c) methodological appropriateness, (d) relevance of evaluated parameters, and (e) coherence between results and stated aims. Each criterion contributed 20% to the overall score. Studies scoring below 80% were excluded from the meta-analysis. Of the 22 full-text articles assessed for eligibility, all met the quality threshold and were therefore included in the meta-analysis. The summarized characteristics and quality scores are presented in (Table 1 and Table S1).

#### 3.1.2. Geographical Distribution and Digital Infrastructure

Approximately 60% of the included evidence derives from high-income countries, predominantly the United States (41.9%), Canada (18.2%), Germany (13.6%), and Australia (9.5%). This distribution underscores a pronounced North–South innovation divide, those risks exacerbating existing disparities in access to digital health technologies if not proactively mitigated (Supplementary Figure S1).

#### 3.1.3. Epistemic Meta-Analysis Outcomes

In this study, we adopt the definition proposed by Ayala et al. [17], who describe meta-analysis as an approach used in research to analyze and synthesize existing knowledge, theories, or findings. The term “epistemic” refers to the examination of knowledge and understanding through the evaluation of underlying assumptions, methodologies, and theoretical frameworks within scientific practice. This approach aims to provide a deeper understanding of the current state of knowledge, highlighting its strengths, limitations, and areas that require further investigation.

**Figure 5:**
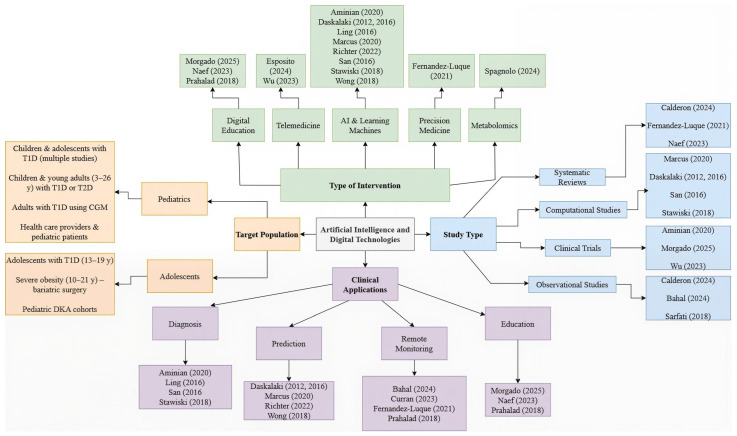
Classification of studies. This figure shows a concept map that classifies the studies according to the type of design, the target population, the type of intervention, and their clinical application. It allows visualization of the methodological and thematic diversity of the included studies, as well as the relationship between technological approaches and clinical purposes, which facilitates the interpretation of the results in relation to the research question [1,2,3,4,10,11,12,14,15,16,18,19,20,21,22,23,24,25,26,27].

**Figure 6:**
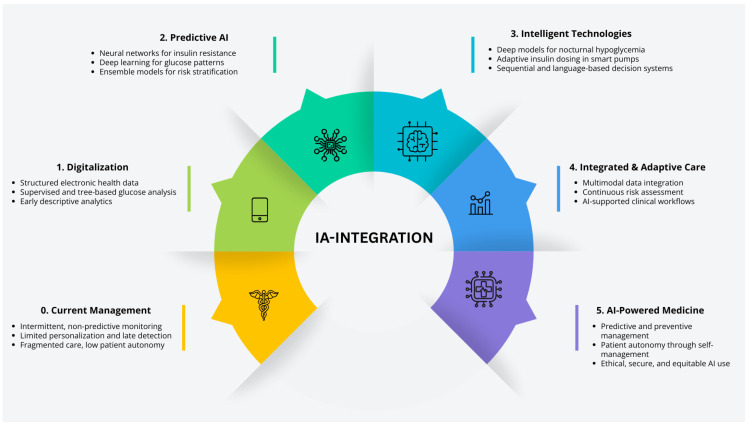
Five phases road map. The transition map represents the transformation from traditional pediatric diabetes care to five phases of enabled AI: personalized management, spotlighting milestones, digitization, new devices, CGM, predictive algorithms, and decision support. These AI applications collectively advance diagnosis, treatment, and monitoring.

**Figure 7:**
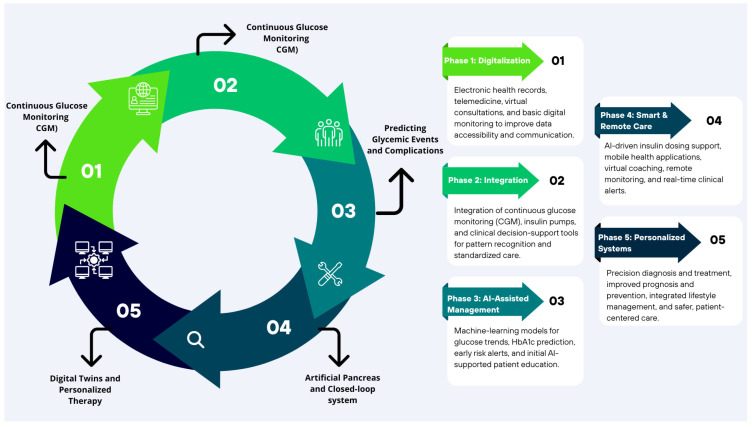
Transition toward digital medicine and artificial intelligence. The figure illustrates the progressive evolution from traditional medicine toward an AI-powered, personalized, and education-supported diabetes care.

In our work, this epistemic meta-analysis is applied to the field of artificial intelligence (AI) in endocrinology, with a particular focus on pediatric diabetes care. The analysis follows these key steps:

1. Identification of relevant papers that address the research question (Table 1)
2. Analysis of the types of tacit and explicit knowledge involved in AI applications for pediatric diabetes care and their interrelationships. (Table 2 and Table 3, and Figure 4 and Figure 5)
3. Identification of existing knowledge gaps. See limitation of the evidence in Section 4.1
4. Examination of knowledge exchange processes within the field of AI and pediatric diabetes care. (Table 3 and Table S2).
5. Generation of novel insights to advance and improve AI applications in pediatric diabetes care (Figure 6 and Figure 7 and see Section 4).

An epistemic meta-analysis can be further enriched through hermeneutical analysis, which constitutes a foundational element of narrative systematic reviews. The key distinction between epistemic meta-analysis and narrative systematic review lies in their methodological approaches. Narrative reviews typically employ qualitative tools, such as ATLAS.ti, along with coding and thematic analysis techniques. However, in general terms, all forms of analysis are epistemic in nature, as they fundamentally rely on the interpretation and organization of knowledge.

##### Study Architecture and Sample Range

The selected studies were organized into four principal categories, as illustrated in (Figure 5): (i) study design, (ii) type of intervention, (iii) technological application, and (iv) target population. This categorization provided an integrative framework for understanding the current landscape of artificial intelligence and digital technologies applied to pediatric endocrinology. Within the study design, the included research encompassed a variety of methodologies—clinical trials [1,10,15,20,22,23], observational studies [10,12,13,16,25], systematic and narrative reviews [10,18,19,27], as well as computational and metabolomic analyses [2,3,12,21]. One pilot study was also identified, reflecting emerging interventions undergoing initial validation. In terms of intervention type, the studies covered a broad spectrum of digital innovations, including machine-learning–based approaches, remote monitoring systems, educational platforms, and diagnostic support tools. Regarding technological application, the predominant focus lay in diabetes management through education, diagnosis, hyperglycemia prediction, and personalized therapy. AI-driven predictive modeling and treatment automation—particularly via wearable devices—were recurrent themes. Finally, target populations were classified into three groups: pediatric cohorts, adolescent-only samples, and mixed populations, including adults, children, and healthcare professionals. This diversity underscores the need for a comprehensive, life-course approach to pediatric diabetes care.

The distribution of study populations, artificial intelligence and digital approaches, reported clinical outcomes, performance metrics, and key methodological limitations across these categories is synthesized in (Table 4).

##### Intervention Typology

The included interventions were categorized into six domains according to their technological foundation, therapeutic objective, and degree of user interaction (Figure 2 and Figure 3):

1. AI-driven prediction and control: Approximately 31.8% of studies employed machine learning or neural network algorithms for glucose forecasting, hypoglycemia prediction, and personalized insulin titration [3,13,16,18,20,22].
2. Monitoring and treatment devices: These included continuous glucose monitoring (CGM), insulin pumps, and hybrid closed-loop (“artificial pancreas”) systems [1,2,10,12,24,26]. The implementation of such devices produced notable improvements in glycemic control, with reduced variability and increased proportions of time within target HbA1c ranges compared with conventional approaches [15,24,26].
3. Digital self-management education: Mobile applications, gamified platforms, and web portals enhanced adherence to treatment by 25–30% and improved self-management empowerment among approximately 60% of participants [4,15,27].
4. Telemonitoring and remote care: Video consultations, asynchronous messaging, and daily data uploads increased the frequency of patient–clinician interactions without extending clinical encounter duration [15,18,21,23].
5. Metabolomic diagnostics: Mass-spectrometry–based approaches identified discriminatory metabolite profiles associated with diabetic ketoacidosis [22], marking an emerging frontier in personalized metabolic medicine.
6. Decision-support dashboards and data visualization: Cloud-based platforms such as Tidepool aggregated device data from multiple sources, facilitating timely responses to glycemic fluctuations and enabling individualized insulin-dosing adjustments [1,16,19,23].

Over half of the studies demonstrated significant improvements in clinical outcomes, including reduced HbA1c levels, fewer hypoglycemic events, enhanced disease management, improved adherence, and greater patient confidence. From a methodological perspective, most studies exhibited a low risk of bias. Common limitations, however, included small sample sizes, heterogeneous evaluation protocols, short follow-up durations, and limited external validation of AI models [24]. Collectively, these findings underscore the potential of AI-driven and automated monitoring technologies as safe and effective tools for optimizing the management of endocrine disorders, particularly diabetes, in pediatric and adolescent populations [1].

##### The Digital Shift in Pediatric Endocrinology: Results Across 22 Studies

A cross-study synthesis of the 22 papers reveals how swiftly pediatric and adolescent diabetes care is shifting toward data-driven, digitally mediated models. Investigators are moving past episodic, visit-based management to test automated monitoring platforms, predictive algorithms, and telemedicine workflows. Computational modeling and algorithm-validated studies dominate, followed by systematic reviews and small pilot trials exploring real-world feasibility. Advanced machine-learning analyses use rich continuous data paired with electronic health records and cloud platforms, which includes CGM traces, insulin-pump logs, and ECG-derived signals (HR, QTc). Nine studies employed AI-based predictive models (neural networks, regression ensembles), while others leveraged continuous physiological monitoring to flag abnormal glucose trajectories and anticipate adverse events [3,12,24].

Clinically, these efforts converge on practical goals: real-time hypoglycemia prediction and prevention; tighter glycemic controls with fewer clinic visits; improved adherence and autonomy; and shared decision-making supported by digital literacy tools [12,22,26]. In four studies, digital systems achieved superior, or at least equivalent, sensitivity and specificity compared with conventional diagnostics, underscoring their readiness for integration into routine care [1,18,24]. Digital health literacy emerged as a decisive moderator of outcomes: families trained to use these technologies managed the disease more effectively and adhered better to therapy [21]. Work centered on education and self-management also exposed persistent communication gaps between patients and clinicians; narrowing that gap is essential for individualized therapy [3,15,23]. New platforms, from educational video games to closed-loop insulin delivery systems and real-time dashboards, mark a clear turn toward automated, personalized, patient-centered care [4,12].

##### Two Paradigms of Digital Care: Platforms vs. Predictive Engines

The 22 studies, summarized across three thematic tables, demonstrate how AI and advanced digital systems are being leveraged against pediatric T1D and T2D. Organized into tables that cover (i) clinical application, (ii) technical function, and (iii) computational methodology, the dataset not only classifies each study but also exposes converging trends, persistent gaps, and openings for future deployment.

Roughly 60% of the studies rely on digital platforms without embedded predictive algorithms. These tools center on continuous glucose monitoring, data visualization (CGM/pump dashboards), patient-family education, and digital-health literacy initiatives [2,4,11,13,20,23,24]. By making glycemic patterns visible and intelligible, they help clinicians, parents, and children adjust therapy collaboratively and communicate more effectively [10,22,24]. Many integrate feedback loops or game-like elements to raise adherence and foster autonomy in adolescents.

The remaining 40% deploy machine- or deep-learning models to tackle harder problems, anticipating hypoglycemia, optimizing insulin dosing, estimating long-term complication risk, or extracting physiological signatures for personalized care [20,22,23,26,27]. These advanced systems most often report meaningful gains in HbA1c, diagnostic accuracy (sensitivity/specificity), and treatment adherence [15,19,23,27]. Several also document improved quality of life and a lighter emotional burden of self-management [4,22]. Methodological rigor, however, is uneven. Validation strategies range from narrative reviews with no clinical testing to trials that are unapproved or simulator-bound [13,18]. Only a subset of models is currently integrated into routine care, leaving a translational gap between innovation and implementation.

The interventions of these studies and classifications are represented in (Supplementary Figure S1); they were grouped into 5 categories, which were determined by type of intervention, clinical application, validation techniques, type of study, and target population.

In short, (Table 2) makes the bifurcation clear: one stream prioritizes user-friendly, educational platforms already easing daily management; the other pursues predictive intelligence that promises precision but still faces regulatory, validation, and deployment hurdles.

It is important to point out that only a minority of studies evaluated AI systems under real-world clinical conditions with external validation. Several predictive models remained confined to simulated environments or retrospective datasets, thus limiting their immediate translational value.

##### Methodological Landscape of AI-Driven Diabetes Tools

Viewed through a technical lens, the 22 studies reveal how pediatric diabetes research is experimenting with a broad toolkit of algorithms, data streams, and validation strategies. Table 2 catalogues these elements-machine-learning type, feature sets, targets, and reported constraints—so the landscape can be read immediately.

Most groups relied on supervised learning to classify states or predict near-term events [1,12,13,19,20,24]. A substantial subset pushed into deep-learning territory: ANNs, CNNs, and RNNs, particularly when modeling complex temporal or physiological signals. Notable innovations fuse physiological knowledge with AI, for example, neural-network insulin-absorption models that sharpen forecast accuracy [21,22].

Rather than foregrounding data provenance, the studies concentrate on how information is organized and learned from. Fixed clinical descriptors, such as age, sex, Hb1Ac, insulin dose, and BMI, are blended with higher-frequency behavioral and physiological rhythms such as activity patterns or circadian variation [11,12,16,26,27]. Handling these heterogeneous, sequential inputs typically pushed investigators toward deeper architectures capable of capturing long-range dependencies.

The models themselves pursue five practical outputs that map neatly onto the clinical pathway: (i) near-term glucose forecasting (nocturnal hypoglycemia, post-prandial spikes) [1,11,13], (ii) automated, individualized insulin dosing via closed-loop/artificial-pancreas systems [3,16,22]. (iii) prediction of long-term complications, retinopathy, nephropathy, and cardiovascular disease using longitudinal clinical histories [13,22,24]. (iv) early diagnostic automation for DKA or insulin resistance through biomarker integration [2,26] and (v) personalized education and psychosocial support tools that strengthen adherence and autonomy [10,23,27].

Despite this sophistication, translation lags. Many models rest on pediatric cohorts smaller than 100, depend on simulators, or lack external validation, which blunts generalizability [2,10,19,22]. Structural barriers, limited connectivity, device cost, and low digital literacy further impede uptake in underserved settings. (Table 2) thus captures both the methodological maturity of current AI work and the parallel need for stronger datasets, ethical and regulatory scaffolding, and equitable infrastructure to carry these systems from prototype to practice.

##### The AI Care Pipeline: Linking Base Data to AI Outputs

(Supplementary Table S2) integrates the key characteristics of every study, raw data streams, algorithmic inputs, and clinical outputs, highlighting how performance rises or falls with the richness and reliability of the underlying data. Taken together, these papers show AI moving pediatric diabetes from guideline-driven averages to physiology-matched, patient-specific therapy.

Across the portfolio, advanced machine-learning systems (e.g., closed-loop “artificial pancreas” algorithms) deliver the clearest gains in glucose forecasting, autonomous insulin titration, and day-to-day adherence [2,14,20,22,24]. Telemedicine layers on top of these engines, extending specialist oversight through virtual consults and continuous remote monitoring; for families in rural or underserved areas, that means fewer clinic visits, lower costs, and real-time dose adjustments [3,11,14,18,20,24].

Education-centered interventions further expand the digital ecosystem. Mobile apps, interactive platforms, and serious games provide tailored coaching on nutrition and activity while offering direct messaging channels for rapid clinician feedback, an approach that bolsters engagement and empowers children and adolescents to self-manage with confidence [12,13,14,18,20,21,24].

The most ambitious studies in the future are trying digital twins that combine clinical, genomic, and metabolic data to model each child’s future risk landscape. Coupled with AI analytics, these models promise to flag retinopathy, nephropathy, or cardiovascular complications years before conventional markers emerge, ushering in a new era of proactive, preventive care [2,14,18,20,22,23,24].

Yet progress is uneven. Small pediatric cohorts, simulator-only testing, and stark disparities in device availability mean that the benefits documented here remain out of reach for many. Robust validation across diverse populations, equitable reimbursement models, and large-scale digital literacy programs are essential if these innovations are to translate into universal gains rather than widened gaps in care.

##### Digital Equity and Ethical Considerations in AI Implementation

Persistent access gaps cast a long shadow over the promise of AI-enabled diabetes care. Most deployments cluster in countries with mature digital infrastructures, such as the United States, Canada, Germany, and Australia, leaving resource-limited regions largely untouched [18]. Even within high-income settings, low digital literacy among children and caregivers can blunt the impact of otherwise sound interventions, especially in socioeconomically vulnerable groups [15,19].

Ethical scaffolding also lags. Clear standards for safeguarding minors’ data, securing meaningful consent, and auditing algorithmic decision-making remain patchy [18,27]. Without deliberate investment in connectivity, user training, and robust governance, AI risks widening rather than narrowing pediatric health disparities. Equitable rollout, grounded in affordability, education, and transparent regulation, must therefore travel in lockstep with technical innovation.

##### The Endocrinology and AI Binomial: Driving Innovation in Modern Medicine

Endocrinology’s data-dense, longitudinal nature makes it ideally suited for AI integration. AI processes high-frequency, multi-modal data to discover latent patterns and generate real-time, patient-specific predictions, supporting earlier and more precise clinical decisions [11,14,22].

Pediatric diabetes exemplifies this synergy. Care hinges on continuous dose adjustment, vigilant monitoring, and sustained educational and behavioral support [1,10,15]. Machine-learning algorithms, deep neural networks, and closed-loop systems (“artificial pancreas”) automate large parts of this workload, tailoring insulin delivery to an individual child’s physiology, reducing human error, and smoothing glycemic variability [2,12,22].

Beyond day-to-day control, AI extends endocrinology into predictive and preventative medicine. Models trained on longitudinal clinical data, augmented with genomic, metabolic, and other ‘omics signatures, can stratify risk and flag complications years before overt clinical expression and even help differentiate endocrine disorders with overlapping phenotypes [21,24,25]. This shift from reaction to anticipation represents a qualitative change in the discipline’s reach and precision.

In sum, the fusion of AI and endocrinology is not merely a technological upgrade; it reframes the care model toward efficiency, equity, and personalization. With appropriate validation, governance, and access, this alliance can consolidate a 21st century standard of care that delivers earlier interventions, fewer complications, and greater autonomy for young patients and their families [3,13,18].

The transition map (Figure 6) describes the five sequential phases in the implementation of new artificial intelligence (AI)-based technologies for diabetes management, depicting the progressive transition over time from the use of basic medical tools to fully digitalized and advanced solutions oriented toward personalized medicine. It reflects the evolution of medical practice through the integration of technological innovations driven by AI models. Each phase highlights the specific application of technologies and algorithms for continuous glucose monitoring (CGM), early prediction of complications, automation of decision-making, and the development of digital health platforms, along with their emerging benefits. This progression demonstrates how these advances enhance the individualization of diagnosis, treatment, and prognosis of diabetes, optimizing the full potential of care in the pediatric population.

##### Artificial Intelligence and Education: Harnessing Metabolic Memory to Redefine Diabetes Management in New Generations

Education remains the authentic alternative to optimize metabolic memory and delay the development or progression of complications and comorbidities, especially in Generation Z children, who are born with integrated technology. Although diabetes mellitus is much more than glucose, continuous glucose monitoring (CGM) teaches them to better integrate the three main tools for control: the meal plan, understanding how the glucose curve behaves not only in relation to the grams and type of carbohydrates but also with fats and proteins and their combination with fruits or vegetables; exercise, identifying how glucose varies depending on physical activity, considering the intensity, type, and duration of the effort; and insulin, using carbohydrate counting programs that allow for a more precise calculation of the prandial dose of ultra-rapid insulin and the basal insulin [5]. With intensive treatment that includes five insulin applications per day, it is possible to achieve control similar to that of insulin pump therapy, although the pump makes the process easier. Artificial intelligence (AI) has emerged as a powerful tool that facilitates education and diabetes management [4], although it can also present failures. For example, in previous generations of insulin pumps, if the cannula became obstructed, the patient believed they were receiving insulin when in fact they were not, which could trigger ketoacidosis. This type of situation opens new perspectives and raises important ethical dilemmas: who is responsible in these cases? The patient, the family, the technology provider, or the physician and the healthcare team? Despite these challenges, thanks to technological innovations, intensive treatment with multiple insulin applications can achieve control comparable to that of the insulin pump, although the latter further facilitates the process. In conclusion, artificial intelligence has transformed from a simple promise to becoming a fundamental tool in the revolution of how we currently manage diabetes, optimizing disease treatment [6]. Powered by education, AI becomes an invaluable ally for self-management and improves the quality of life of patients, especially those of new generations, who grow up with technology. Continuous glucose monitoring and AI facilitate the integration of the three pillars of metabolic control: nutrition, exercise, and insulin, optimizing diabetes treatment and opening new perspectives in its management (Figure 7) [4,15,28].

##### Data Privacy, Integration, and User-Centered Outcomes in AI-Enabled Diabetes Care

The implementation of artificial intelligence (AI) and digital platforms for diabetes care raises considerations about privacy in data use and its integration in diagnostic and predictive algorithms. In all the evaluated studies, the implementation of AI is based on the use of real-time and continuous data. These data are collected from portable monitoring devices, mobile applications, and tablets that store information in the cloud through electronic medical records [1,11,12]. The collection of these data provides the algorithms and allows them to feed and develop responses for personalized clinical decisions according to the evaluated data. Therefore, it questions the exposure to privacy and security risks of these sensitive patient health data.

The exchange of data between health personnel, databases, technology providers, and patients is not addressed in detail in the ability to monitor ethically and continuously the use of this data by those who make use of AI [18,27]. The lack of law initiative for transparent data regulation and their regulated and secure use complicates the implementation of AI-driven algorithms in decision-making, a process that still generates mistrust [4].

The integration of AI into daily clinical practice presents a challenge in the organization of systems and the ability to maintain the privacy of data integrated within algorithms and platforms, which limits its implementation to current management in a secure and sustained manner [1,11].

These findings show that, despite the innovative opportunities AI offers to improve patient personalization and empowerment, its clinical impact depends on adequate data protection, transparent governance, and adequate integration through the training of health personnel and continuing follow-up.

##### Ethical Foundations and Bioethics Governance in the Care of Pediatric Diabetes with Artificial Intelligence (AI)

**Figure 8:**
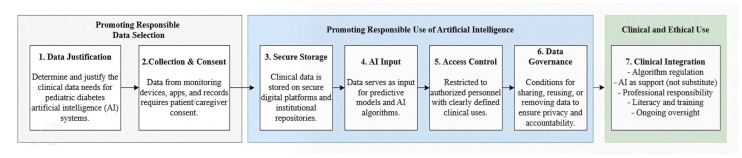
Conceptual framework for the responsible and ethical use of artificial intelligence in pediatric diabetes care. The framework describes a seven-stage process governing the ethical and clinical use of artificial intelligence in pediatric diabetes care. Stage 1 (Data Justification) focuses on identifying and justifying the specific clinical data required for AI-based applications. Stage 2 (Collection and Consent) ensures that data from monitoring devices, digital applications, and clinical records are collected only after obtaining informed consent from patients and caregivers. Stage 3 (Secure Storage) involves the protection of clinical data within secure digital platforms and institutional repositories. Stage 4 (AI Input) represents the use of curated data as input for predictive models and artificial intelligence algorithms. Stage 5 (Access Control) restricts data availability to authorized personnel and clearly defined clinical purposes. Stage 6 (Data Governance) establishes policies for data sharing, reuse, and removal to ensure privacy, transparency, and ethical accountability. Finally, Stage 7 (Clinical Integration) regulates the implementation of AI in clinical practice, emphasizing its role as a decision-support tool rather than a replacement for medical judgment. This stage also highlights professional responsibility, digital literacy, training, and continuous ethical oversight.

The integration of AI into pediatric diabetes care requires the development of an ethical framework that focuses not only on technical performance but also on clinical efficiency (Figure 8). Among the main concerns are patient autonomy, transparency of data use, responsibility, and equitable use of technology [11,12,27].

From an ethical and clinical perspective, patients should be perceived as unique individuals who cannot be reduced to algorithmic data or output [17]. Children and adolescents with diabetes represent a particularly vulnerable population due to their difficulty understanding informed consent, exposure to pilot studies and digital monitoring, and reliance on their caregivers for decision-making. This also underlines the need to focus the development and implementation of AI tools in human care, ensuring their alignment with the principle of beneficence and non-maleficence [11,15,18].

##### Artificial Intelligence, Clinical Judgment, and Doctor-Patient Relationship

The implementation of artificial intelligence poses new challenges for the doctor-patient relationship by introducing algorithms aimed at automatizing clinical decision-making and predictive analysis. While these tools can improve the safety and accuracy of therapeutic management, they should not replace clinical judgment or human decision-making, as all AI-driven models and systems require continuous monitoring, constant updating, and professional supervision.

In the context of glucose prediction and therapeutic insulin adjustment, the medical team retains clinical responsibility for interpreting the results generated by the algorithms and making appropriate adjustments to prevent acute and chronic complications [19,22,24].

Artificial intelligence also functions as a support tool but does not replace clinical reasoning in any of the specialties that make up the multidisciplinary team, including nutrition, physical activity, mental health, and comorbidity management [4,12,18].

##### Impact of Artificial Intelligence in the Management of Type 1 and Type 2 Diabetes in the Pediatric Population

Outside of a uniform impact, the results show that artificial intelligence (AI) is transforming the management of pediatric diabetes into two different types of diabetes (as summarized in Table 3) [1,2,3,4]. In type 1 diabetes (T1D), AI works as a tool for monitoring and continuous follow-up in real-time and therapeutic automation to promote the autonomy of patients [10,11,14,22,26,29]. In this context, some studies have reported modest but clinically consistent improvements in the results of glycemic control. Some enhancements included are a reduction of HbA1c ranging from 0.2 to 0.3% (<7%), an increase in time in range (TIR 70–180 mg/dL) of approximately 5 to 15 percentage points, and a reduction in the frequency or variability of critical hypoglycemia events when AI-based tools are safely and guidedly integrated and continuously monitored [24,25,27].

Meanwhile, in type 2 diabetes mellitus (T2D), the role of AI focuses on risk prediction and prevention with both an approach to early stratification of metabolic risks and cardiovascular studies with a longitudinal analysis of the collected clinical data. It uses its educational applications and platforms for effective lifestyle change [12,13,15,16,18]. Nonetheless, studies demonstrate that a great clinical impact depends on the adherence of patients and the directed interventions of the multidisciplinary medical team [19,21].

The potential of AI depends not only on how sophisticated or innovative it is, but also on its proper integration and the digital literacy of users and doctors. T1D benefits from automation and preventive control to avoid critical events that reduce hospitalizations, and T2D benefits from the anticipation of risks and prevention of long-term complications. In both scenarios, methodological changes are still required, highlighting the need to develop prospective, long-term studies that confirm these findings in the pediatric population [18,27].

This table synthesizes the distinct and shared clinical roles of artificial intelligence across pediatric type 1 and type 2 diabetes. It highlights how AI-driven approaches align with differing clinical objectives, data requirements, and care priorities. Rather than providing an exhaustive description, the table offers a conceptual framework to contextualize the diverse applications of AI according to disease-specific needs and stages of clinical integration.

The following table summarizes the different populations, artificial intelligence and digital methods, and clinical outcomes reported in the studies included in this review. The analysis shows differences in performance metrics and how advanced the methods are, while also highlighting common limitations related to study design, the characteristics of the groups studied, and how the findings are verified. The table highlights the clinical potential of AI-based methods while also showing the structural challenges that currently limit broad comparisons and practical use.

## 4. Discussion

Artificial intelligence (AI) is no longer an intriguing add-on to pediatric diabetes practice; it is quietly rewriting the entire playbook. The 22 high-quality studies we reviewed [1,2,3,4,10,11,12,13,14,15,16,18,19,20,21,22,23,24,25,26,27,28] sketch a clear narrative arc (Figure 2). It begins in the clinic, where machine-learning image classifiers now screen retinal photographs with a level of sensitivity and specificity that once required a specialist’s eye [14], while metabolomics-based models flag children teetering on the brink of ketoacidosis long before biochemical chaos erupts [22]. Simultaneously, non-invasive wearables and continuous-glucose monitors stream data into predictive engines that learn each child’s rhythm and whisper warnings of an impending rise or plunge hours before symptoms appear.

Those same data streams feed the “thinking” pumps and closed-loop systems that have redefined therapy. Instead of the rigid, clinician-programmed basal-bolus schedules of the past, adaptive algorithms adjust doses in real-time, trimming glycemic peaks, flattening valleys, and sparing families the dread of nocturnal hypoglycemia [2,10,15,26]. The result is not merely better numbers on a glucometer but a palpable shift in agency: children and parents increasingly move from crisis management to confident, data-supported self-care. This is reflected in the findings from (Table 1), this table summarizes the clinical improvement observed with the implementation of AI.

AI’s predictive capabilities extend to long-term complications. Physiological-signal models can predict hypoglycemia minutes in advance [11,20]; by tracking longitudinal electronic-health-record patterns, other algorithms forecast the onset of retinopathy or nephropathy up to a decade before traditional markers would sound the alarm [23]. Layered on top of this predictive scaffolding are telemedicine platforms, gamified apps, and conversational agents that translate complex analytics into engaging, age-appropriate coaching, improving adherence and stretching specialist expertise to remote or underserved communities at lower cost (Figure 6 and Figure 7) [3,12,13,21,24].

Of course, the story is still being written. Many of these promising tools were tested in small, homogeneous cohorts, over short follow-up periods, and with limited external validation, raising questions about how well they will perform in the real-world heterogeneity of low-resource settings where internet bandwidth, device availability, and digital literacy remain uneven [12,15,27]. The next chapter, therefore, must feature broad, multicenter trials embedded in diverse socioeconomic contexts, alongside parallel work on regulatory guardrails, clinician training, and reimbursement pathways [12,13,18,20].

### 4.1. Limitations of the Evidence

This systematic review has certain limitations that must be considered in the interpretation of results. In the first instance, all included studies met the PRISMA quality criteria, but these have different methodologies implemented. Among this variety, observational studies, pilot studies, systematic reviews and narratives, computational model studies, and a limited number of clinical trials can be found [2,13,27]. This difference in methodology made it impossible to make a quantitative comparison between these studies, limiting the results to be generally concluded (Table 4).

Another important limitation is that the AI interventions in the studies were evaluated in small cohorts, short test periods, and machine learning-controlled or simulated environments. Several prediction models were retrospectively validated, with very limited external validation in selected populations [10,22,24,26]. Therefore, it is concluded that the final performance of AI in diabetes management is still uncertain, especially in vulnerable populations such as pediatric.

Third, the implementation of artificial intelligence had large variations from AI-driven tools. Some studies implemented models based on machine learning or neural network implementation, while others relied on the application of digital platforms for the implementation of telemedicine or technologies with an educational focus without the use of prediction algorithms [1,12,15]. This variability makes direct comparisons difficult, highlighting the need for more explicit classifications that can categorize AI-driven tools more clearly for universal implementation in today’s health systems.

Another important limitation is the geographical and socio-economic variability, which was very uneven since most studies were carried out in developed countries with an economic capacity to acquire these tools more effectively due to their digital infrastructure. Thus, it limits the evaluation of its effectiveness and equitable implementation in low- and middle-income countries [18,27]. Even in these economically developed regions, the difficulties for access to these devices and adequate digital literacy have been mentioned among the main obstacles for the implementation of AI in the health sector [19,20].

Conclusions are limited because the results of the studied methodologies were not evaluated in the long term to assess a sustained reduction of complications and their cost-effectiveness for the health sector. This highlights the need to develop much broader studies with long-term validation in order to better define the role of AI in diabetes care for children and adolescents [3,25].

### 4.2. Future Directions and Clinical Readiness of Artificial Intelligence in Diabetes Care

The findings of this review highlight the current application of artificial intelligence (AI) in diabetes care and the key tendencies that are likely to define its future clinical integration. In the studies reviewed and included, the adoption of AI is in a phase of progressive acceleration, driven mainly by continuous glucose monitoring systems, insulin delivery technologies, wearable devices, telemedicine platforms, telemonitoring, and a growing capacity to process large volumes of data [1,11,12,27]. There is no uniform reporting of adoption rates, as these vary mainly by region. Nevertheless, the overall trajectory suggests a gradual, though not yet sustained, expansion of AI-driven tools, particularly in settings with consolidated digital infrastructures [18,27]. However, quantitative estimates of AI adoption remain limited and heterogeneous across healthcare systems, making direct comparisons between regions and social contexts difficult.

Regarding clinical readiness, current evidence indicates that AI systems are at different stages of adoption and maturity. Some applications, such as closed-loop automated insulin delivery systems, real-time glucose prediction models, and hypoglycemia detection algorithms, are still under development and have not yet been fully incorporated into routine clinical practice, particularly in the management of type 1 diabetes in pediatric populations [10,24]. This gap is more evident in pediatric populations than in patients with type 2 diabetes, such as adolescents and future adults. In these cases, preventive interventions oriented toward lifestyle modification predominate but still lack robust prospective validation [14,19]. Several challenges remain in the determination of the appropriate pace and direction of AI adoption in diabetes care. These challenges are largely related to methodological diversity across studies, including small sample sizes, short follow-up periods, and the lack of standardized performance evaluation metrics, which limit comparisons across investigations. Additional barriers include limited external validation, lack of algorithmic transparency, regulatory uncertainty, and unequal access to digital infrastructures. If not adequately addressed, these factors may further exacerbate existing health inequalities [22,26]. The effective implementation of AI-based tools also depends on the level of digital literacy among patients, families, and healthcare professionals, as well as on their seamless integration into supervised clinical workflows that are applicable across different specialties within multidisciplinary care teams [1,18].

Looking forward, diabetes care is expected to evolve toward integrated ecosystems centered on the patient, incorporating artificial intelligence to combine real-time physiological data, electronic health records, lifestyle information, and emerging biomarkers (such as metabolomic profiles) [14,21]. This shift aims to move diabetes management from a reactive approach toward predictive, preventive, and personalized care with potential long-term benefits for life quality and metabolic outcomes. Achieving this transition will require coordinated methodological advances, ethical governance frameworks, and equitable implementation strategies to ensure that technological innovations are safe, clinically meaningful, and accessible to diverse populations [27,28]. Together, these tendencies help clarify the pace for AI adoption, the current level of clinical readiness, and the main limitations affecting its integration into routine diabetes care.

## 5. Conclusions

The integration of artificial intelligence into pediatric endocrinology has transitioned from a conceptual aspiration to an established and expanding clinical reality. Current evidence demonstrates that AI enhances metabolic control, facilitates early complication prediction, and augments clinical decision-making, while simultaneously empowering patients through educational and self-management technologies. Collectively, these developments are fostering a new paradigm in diabetes care—one that is predictive, preventive, personalized, and participatory.

Nevertheless, the consolidation of AI within clinical practice necessitates the resolution of several critical challenges: the execution of robust multicenter validation studies, the establishment of comprehensive data governance and storage regulations, the formulation of ethical and legal frameworks, and the systematic training of healthcare professionals in digital and computational literacy.

Artificial intelligence has already begun to rewrite the script of pediatric diabetes care. Closed-loop “artificial-pancreas” systems and adaptive insulin pumps now trim hypoglycemic episodes and lighten the daily therapeutic load, all while keeping children in tighter time in range than traditional regimens could manage [10,22,23]. In parallel, deep learning models that track physiological signals, QTc dynamics, heart rate variability, and more can sense the tremor of an impending glycemic swing before it surfaces, adding a silent, non-invasive layer of protection [11,12,27]. Hardware is only half the story: digital health platforms, chatbots, gamified apps, and telemedicine portals translate complex analytics into engaging guidance, strengthening adherence, and placing families at the center of day-to-day decision-making [1,15,23].

However, the promise must be validated. Many innovations have been evaluated in narrow cohorts, leaving questions about performance across diverse populations and settings. Bridging the evidence gap requires large multicenter trials and concerted efforts to address infrastructure and digital literacy divides. Clinicians must be equipped to interpret AI outputs and integrate them into shared decision-making.

If those pieces fall into place—rigorous validation, inclusive deployment, and robust ethical safeguards—AI can propel pediatric endocrinology from episodic, glucose-focused care toward a continuous, predictive, and truly patient-centered model, delivering earlier interventions, fewer complications, and greater autonomy for every child and family living with diabetes.

## Figures and Tables

**Table 1:** PRISMA evaluation—this table represents the qualitative evaluation of the studies selected through the PRISMA methodology. this evaluation allowed us to estimate the scientific importance and the level of contribution.

Study Reference (Author, Year, Country)	Clear Objectives	Research Question	Methodology	Outcome Parameters	Reported Results	Quality Score
(Wong et al., 2018, USA) [1]	Evaluate a software platform to integrate data from diabetes devices. 20%	Does the software improve data integration and accessibility for diabetes management? 20%	Pilot study of 15 healthcare with the usage of a platform. 20%	Frequency of data references, user, and patient satisfaction. 15%	No increase in data load time or consultation. 20%	95%
(Bahal et al., 2024, USA) [2]	Evaluate advances in diabetes management with new technological developments. 20%	What are the recent advances in managing type 1 diabetes in children and adolescents? 20%	Literature reviews of recent advancements in diabetes. 20%	Effectiveness of glucose control tools such as insulin pumps and continuous glucose monitors. 20%	Improved blood glucose control. 20%	100%
(Sarfati et al., 2018, New Zealand) [3]	Evaluate the impacts of BetaMe on diabetes control. 20%	Can digital intervention improve glucose control in children and adults with diabetes? 20%	Randomized trial for 6 months with a control group. 15%	HbA1c levels, patient weight, and adherence to treatment regimens. 20%	Significant reduction in HbA1c levels and improved patient health outcomes. 20%	95%
(Curran et al., 2023, Canada) [4]	Evaluate the impact of a telemedicine platform for remote monitoring in pediatric diabetes care. 15%	Can artificial intelligence improve monitoring and treatment for pediatric diabetes? 20%	Longitudinal observational study on the effectiveness of a telemedicine platform. 20%	Reduction of HbA1c levels, enhanced patient engagement, and remote monitoring effectiveness. 15%	Reduced blood glucose and improved patient satisfaction. 15%	85%
(Richter et al., 2022, USA) [14]	Develop methods to predict surgical outcomes for bariatric surgery. 20%	How can data assimilation models improve prediction accuracy for bariatric surgery outcomes? 20%	Use of mechanistic models to predict surgical outcomes. 20%	Prediction accuracy of bariatric surgery outcomes. 20%	The model predicted post-surgical glucose levels with high accuracy. 15%	95%
(San et al., 2016, Australia) [10]	Develop a deep learning framework for hypoglycemia detection in children with type 1 diabetes. 20%	Can deep learning models improve hypoglycemia detection for type 1 diabetes? 20%	New deep learning models using patient data to predict hypoglycemia. 20%	Model performance in predicting hypoglycemic events. 20%	Hypoglycemia detection accuracy improved significantly using deep learning models. 20%	100%
(Prahalad et al., 2018, USA) [11]	Analyze the impact of diabetes technologies on supporting care. 10%	How can digital technology improve patient education for diabetes care? 20%	Quantitative analysis of digital technology impact on patient care. 20%	Patient engagement, education completion rates, and glucose control metrics. 15%	Positive patient outcomes with digital educational tools. 20%	85%
(Fernandez-Luque et al., 2021, Germany) [12]	Explore the health impact of digital interventions for diabetes education. 20%	How can digital platforms improve the health of people with diabetes? 20%	Quantitative and qualitative study of digital education platforms. 15%	Blood glucose levels and quality of life after using educational tools. 20%	HbA1c levels improved significantly in intervention groups. 20%	95%
(Nkhoma et al., 2021, Taiwan) [13]	Effectiveness of digital interventions for improving diabetes education in type 1 and 2 diabetes patients. 20%	How do digital interventions impact diabetes education and self-management? 20%	Systematic review of digital educational interventions for diabetes self-management. 20%	Impact on patient education outcomes, including adherence to lifestyle changes. 20%	Digital education reduced HbA1c and improved self-management skills. 20%	100%
(Morgado et al., 2025, Brazil) [15]	Map the scientific evidence supporting educational technologies developed for families and children with type 1 diabetes. 15%	What are the feasible educational technologies for children and their families with type 1 diabetes? 20%	Systematic review of data from JBI database guidelines. 15%	Efficiency of educational technologies in patient education. 15%	Educational technologies are identified as effective but lack long-term evaluation. 15%	80%
(Wu et al., 2023, China) [16]	Evaluate the impact of the AI-HEALS system on glucose self-management and control in type 2 diabetes patients. 20%	Does AI-HEALS improve glucose control in type 2 diabetes patients? 20%	Mixed-methods study with randomized controlled trial and qualitative interviews. 10%	HbA1c, treatment adherence, and lifestyle changes. 20%	Improved glucose control in the intervention group. 20%	90%
(Naef et al., 2023, Germany) [18]	Evaluate the impact of digital health interventions on adolescents with type 1 diabetes. 20%	Can digital interventions improve the health of adolescents with type 1 diabetes? 20%	Systematic review following PRISMA guidelines. 20%	Use of social networks, communication with health professionals, and self-management. 15%	Use of social networks improved communication with health professionals by 10%.	85%
(Marcus et al., 2020, Israel) [19]	Develop a machine learning model for predicting blood glucose levels in type 1 diabetes. 20%	Can machine learning models improve blood glucose predictions in type 1 diabetes? 20%	Analysis of continuous glucose monitor data using four machine learning models. 15%	Model accuracy, false-positive and false-negative rates. 20%	Precise model prediction with reduced false-positive rates. 15%	90%
Calderon Martinez et al., 2024, México) [20]	Compare the efficacy of insulin pumps in managing daily glucose fluctuations in children with type 1 diabetes. 20%	Do insulin pumps show advantages over injections in managing diabetes in children? 20%	Systematic review and meta-analysis of comparative studies. 20%	HbA1c, hypoglycemia episodes, quality of life. 20%	Insulin pumps showed advantages in glucose control and hypoglycemia episodes. 15%	95%
(Spagnolo et al., 2024, Canada) [21]	Identify metabolic patterns related to diabetic ketoacidosis in children. 20%	What metabolic patterns are associated with pediatric diabetic ketoacidosis? 20%	Metabolomics analysis of plasma samples from pediatric patients. 20%	Metabolite levels, clinical incidence of ketoacidosis. 15%	65 metabolites were significantly altered in ketoacidosis patients. 20%	85%
(Daskalaki et al., 2016, Switzerland) [22]	Explore the feasibility of a model-free learning approach for controlling insulin in type 1 diabetes. 10%	Can a machine learning model improve insulin administration accuracy for type 1 diabetes? 20%	Development of a reinforcement learning algorithm evaluated in an FDA-approved simulator. 20%	Time in range, hypoglycemia reduction, and FDA-approved simulator performance. 20%	The algorithm maintained normoglycemia 95% of the time with minimal hypoglycemia. 20%	90%
(Stawiski et al., 2018, Poland) [23]	Develop an artificial neural network model to estimate insulin resistance in children with type 1 diabetes. 20%	Can an artificial neural network model predict insulin resistance in children with type 1 diabetes? 20%	Clinical data analysis and modeling using artificial neural networks and multivariable regression. 20%	Insulin resistance, glucose levels, clinical data. 15%	The model showed high precision in predicting insulin resistance, improving traditional methods. 20%	95%
(Ling et al., 2016, Australia) [24]	Develop a non-invasive monitoring system for hypoglycemia in children with type 1 diabetes using machine learning. 20%	Can a machine learning-based non-invasive monitoring system detect hypoglycemic episodes in children? 20%	Machine learning analysis and ECG-based physiological signal analysis in children with type 1 diabetes. 20%	Heart rate, interaction of ECG, QT intervals, and blood glucose. 20%	The machine learning-based system successfully detected hypoglycemic episodes with high precision. 20%	100%
(Aminian et al., 2020, USA) [25]	Develop predictive models to estimate the long-term risk of organ complications in type 2 diabetes. 20%	Can predictive models estimate long-term complications risk in type 2 diabetes. 20%	Data analysis from patients with bariatric surgery and conventional treatment using machine learning. 15%	Mortality, cardiovascular disease, kidney failure, and metabolic complications. 20%	The model predicted complications risk with high accuracy, aiding decision-making. 20%	85%
(Daskalaki et al., 2012, Switzerland) [26]	Develop adaptive models in real-time for predicting glucose profiles in type 1 diabetes patients. 20%	Can real-time adaptive models predict glucose levels in type 1 diabetes? 20%	Comparison of autoregressive models and neural networks for glucose prediction. 20%	Model precision, sensitivity, and individual response time. 10%	Neural networks outperformed traditional models in glucose prediction accuracy. 20%	90%
(Esposito et al., 2024, Italy) [27]	Evaluate the impact of telemedicine on managing pediatric type 1 diabetes, exploring its benefits and challenges. 20%	Can telemedicine improve diabetes management in children with type 1 diabetes? 20%	Systematic review of studies on telemedicine’s impact on pediatric diabetes management. 20%	HbA1c, frequency of consultations, patient, and provider satisfaction. 15%	Telemedicine showed benefits in accessibility and monitoring but mixed results in glucose control. 20%	95%

**Table 2:** Implementation, learning models and validation of digital platforms: This table summarizes the use of artificial intelligence (AI) in diabetes management, incorporating both the digital tools that employ this capability, and the learning models used in these technologies. Continuous glucose monitoring platforms, automated systems for insulin delivery and mobile applications, as well as their implementation status, validation, and licensing, are highlighted. Several learning models (supervised, deep, and regression) are also presented to predict glucose levels, modify insulin doses, and improve patient follow-up. It has information on the accuracy, sensitivity, and specificity of these models, as well as obstacles to implementation that may arise, such as access barriers and technological infrastructure in different areas.

Author, Year	AI Model	Classes of Machine Learning	Methods	Algorithms	Target Disease	Characteristics	Limitations	Validation	Model Application	Model Validation	Big Scale Implementation	Software/Server
(Wong et al., 2018, USA) [1]	Digital Health	N/A	Data visualization and integration	N/A	Type 1 diabetes (T1D)	Data from insulin pumps, CGM, and other devices	Small sample size, pilot phase	Pilot study with 15 healthcare professionals	Integration and visualization of data from glucose meters, insulin pumps, and CGMs for managing T1D in children.	The study involved 15 healthcare professionals managing T1D children over 6 months.	Limited use with potential for expansion	Web platform Tidepool. URL: https://www.tidepool.org/ (accessed on 15 February 2025).
(Bahal et al., 2024, USA) [2]	Digital Health	N/A	Insulin delivery and CGM monitoring	Closed-loop insulin pump systems	T1D in children	Clinical glucose data, insulin dosage	Access to technology in low-resource settings	Narrative review of technological advances	CGM systems and hybrid insulin pumps	Narrative review of current innovations	In clinical use; pending wider availability in low-resource countries	CGM, insulin pumps, and hybrid insulin delivery systems. MySug—N/A Glooko—N/A Dexcom G6 App—N/A
(Sarfati et al., 2018, New Zealand) [3]	Digital Health	N/A	Web/mobile behavior change support	N/A	T2D, Prediabetes	Self-reported behavior and health indicators	Long-term impact not yet assessed	Randomized controlled trial	Self-management and management of T2D and prediabetes via web and mobile platforms.	Randomized clinical trial with 430 participants	Not yet implemented; potential for primary care	BetaMe platform. License: Creative Commons Attribution 4.0 URL: http://www.melonhealth.com/ (accessed on 15 February 2025).
(Curran et al., 2023, Canada) [4]	Digital Health	N/A	Policy analysis and global health overview	N/A	Type 1 Diabetes (T1D)	Global access to care, insulin availability	Descriptive analysis, not data-driven	Literature and IDF-based review	Study on the incidence and increase of T1D in children and adolescents, focusing on low-resource populations	The narrative review focused on global health, including IDF (International Diabetes Federation) data	Provides recommendations to improve LMICs (low- and middle-income countries); not yet implemented at large scale	Orbis International Cybersight. https://cybersight.org/ (accessed on 15 February 2025) License: CC BY 4.0
(Richter et al., 2022, USA) [14]	Machine Learning	Supervised Learning	Glycemic prediction with data assimilation	Mechanistic modeling + machine learning integration	T2D post-bariatric surgery in adolescents	Clinical variables and metabolic data	Small cohort, not validated externally	Retrospective analysis of adolescent cohort	Predicting glycemic levels in adolescents with T2D who are undergoing bariatric surgery.	Use clinical data from adolescents with severe obesity and T2D through retrospective analysis.	Not implemented yet, under evaluation	No specific software platform, server, or version was reported.
(San et al., 2016, Australia) [10]	Deep Learning	Deep Learning	ECG signal classification	Deep Belief Network (DBN)	T1D–Hypoglycemia detection	ECG signal features: HR, QTc	Small sample size (15 children), experimental	Clinical study with 15 pediatric participants	Hypoglycemic states in children with T1D are detected using ECG (HR and QTc).	Validation with 15 children through nocturnal monitoring	Not yet implemented at scale	No specific software platform, server, or version was reported.
(Prahalad et al., 2018, USA) [11]	Digital Health + Big Data Analytics	N/A	Review of digital health outcomes	N/A	Type 1 Diabetes (T1D)	Patient-reported outcomes, clinical data	Heterogeneous sources, not a meta-analysis	Narrative review of clinical studies	Clinical and psychosocial support in adolescents with T1D, improving glycemic control and quality of life	Narrative review of clinical evidence and device use experiences	Mentions use of some technologies, though access gaps remain	No specific software platform, server, or version was reported.
(Fernandez-Luque et al., 2021, Germany) [12]	Digital Health + AI	AI and Digital Health	Precision medicine approach	Personalized feedback algorithms	Pediatric endocrine disorders (incl. T1D)	User interaction data, digital health metrics	Implementation barriers in clinical settings	Narrative review with proposed models	Diabetes prediction in pediatric endocrinology	Narrative review with integrative proposals	In the development phase, it requires more digital infrastructure	Adhera Health platform easypod™ connect (web-based adherence monitoring platform) URL: https://www.easypodconnect.com/ (accessed on 15 February 2025)
(Nkhoma et al., 2021, Taiwan) [13]	Digital Health	N/A	Digital education platform review	Multiple (platform-dependent)	T1D and T2D	Educational content, user engagement metrics	Methodological diversity in studies	Systematic review and meta-analysis	Digital education to improve self-management of T1D and T2D	Systematic review and meta-analysis of digital interventions	Tools are limited due to heterogeneity	N/A
(Morgado et al., 2025, Brazil) [15]	Digital Health	N/A	Content development and validation	N/A	Type 1 Diabetes (T1D) in children	Educational materials, expert input	Localized study, no predictive modeling	Content validation with professionals and families	Education for families of children with T1D for disease management	Methodological study validated by professionals and users	Used in Brazilian primary care institutions	N/A
(Wu et al., 2023, China) [16]	Artificial Intelligence	Artificial Intelligence	AI-guided health education	AI-HEALS platform	Type 2 Diabetes (T2D)	User interaction and behavioral data	Protocol stage; not yet validated	Planned mixed-methods study	Personalized education for T2D self-management (AI-HEALS)	Quantitative study and qualitative interviews	Not yet implemented	AI-HEALS Platform: WeChat service platform–“PKU Diabetes Butler” URL: https://www.wechat.com/ (accessed on 15 February 2025).
(Naef et al., 2023, Germany) [18]	Digital Health	N/A	Digital intervention review	Multiple platforms	Type 1 Diabetes (T1D) in adolescents	Health literacy and engagement indicators	Heterogeneity in design and outcome measures	Systematic review	Digital interventions to improve health literacy in adolescents with T1D	Systematic review of studies in adolescent populations	Not yet implemented, high potential	N/A
(Marcus et al., 2020, Israel) [19]	Machine Learning	Supervised Learning	Glucose prediction	N/A	Type 1 Diabetes (T1D)	Clinical glucose data from hospital patients	Model not yet deployed in clinical practice	Retrospective clinical data	Blood glucose level prediction	Clinical data from Sourasky Medical Center	Not implemented	N/A
Calderon Martinez et al., 2024, México) [20]	Digital Health	N/A	Comparative effectiveness analysis	N/A	Type 1 Diabetes (T1D)	Outcomes from pump vs. multiple daily injections	Access and variability in study populations	Systematic review and meta-analysis	Comparison of glycemic control between insulin pumps and daily injections in children with T1D	Systematic review and meta-analysis in the pediatric population	Access to pumps varies by country	Continuous insulin infusion technology
(Spagnolo et al., 2024, Canada) [21]	Machine Learning	Supervised Learning	Classification	Support Vector Machine (SVM), clustering	DKA in pediatric patients	Metabolomic profile via NMR/mass spec	Small sample, exploratory study	Case-control study (*n* = 34)	Identification of metabolic patterns in pediatric patients with diabetic ketoacidosis	Case-control study with 34 pediatric patients	Not clinically implemented	Mass spectrometry (Molecular Medicine—open access)
(Daskalaki et al., 2016, Switzerland) [22]	Machine Learning	Reinforcement Learning	Control optimization	Actor-Critic algorithm	Type 1 Diabetes (T1D)	Simulated patient data from the FDA simulator	Simulation only; needs real-world validation	FDA-approved T1D simulator	Personalized optimization of insulin infusion in T1D patients	Validation with an FDA-approved T1D simulator	Not clinically implemented; experimental phase with potential for artificial pancreas	T1D Simulator (FDA-approved)
(Stawiski et al., 2018, Poland) [23]	Artificial Neural Network (ANN)	Supervised Learning	Regression estimation	Artificial Neural Network (ANN), MARS	T1D—Insulin resistance	Clamp test data, 315 children	Model in development phase	Train/test split on collected clinical data	Estimation of insulin resistance in children with T1D	Clinical data from a pediatric population of 315 children	Still in the research phase with possible clinical use	NIRCa calculator based on ANN
(Ling et al., 2016, Australia) [24]	Machine Learning	Supervised Learning	Classification	Extreme Learning Machine (ELM)	Type 1 Diabetes (T1D)—Hypoglycemia	ECG signals during nighttime monitoring	Small dataset (16 children), not deployed	Clinical signal dataset	Non-invasive monitoring for hypoglycemia using ECG signals	Pediatric evaluation in 16 children with nocturnal physiological signals	Not yet commercially available; experimental model	ELM-based system using ECG. License: Rights reserved by Elsevier. URL: https://apps.konsta.com.pl/app/gdr/ (accessed on 15 February 2025)
(Aminian et al., 2020, USA) [25]	Machine Learning	Supervised Learning	Risk prediction	Random Forest	Type 2 Diabetes (T2D) with/without surgery	Clinical history, BMI, outcomes over 10 years	Internal validation only; needs external validation	Large retrospective cohort (*n* > 13,000)	10-year risk prediction of complications in T2D patients with or without metabolic surgery	Internal validation with 5-fold cross-validation in over 13,000 patients	Web and mobile application developed for IDC Risk Scores	Clinical platform for web and smartphones. License: Diabetes Care (restricted access)
(Daskalaki et al., 2012, Switzerland) [26]	Artificial Neural Network (ANN)	Supervised Learning	Time series prediction	AR, ARX, ANN	Type 1 Diabetes (T1D)	Glucose + insulin time-series data	Early-stage model, lab-based validation	In silico evaluation with patient data	Personalized prediction of glycemic profiles and hypoglycemic episodes	Validation with real-world data	Not clinically implemented	N/A
(Esposito et al., 2024, Italy) [27]	Digital Health	N/A	Narrative synthesis of telemedicine outcomes	N/A	Type 1 Diabetes (T1D)	Studies on pediatric care during COVID-19	Narrative format, lack of quantitative metrics	Multiple pediatric study reviews	Monitoring and management of pediatric T1D patients via telemedicine	Narrative review of multiple studies, including pediatric trials during COVID-19	Implemented during the pandemic; growing use, long-term studies needed	N/A

N/A: Not Applicable (Version of the software was not reported in the original study.).

**Table 3:** Differential clinical roles of artificial intelligence in pediatric type 1 and type 2 diabetes.

Aspect Compared	Type 1 Diabetes (T1D)	Type 2 Diabetes (T2D)	Shared Features (T1D & T2D)	Key Differences in Clinical Needs
Primary clinical objective	Real-time glycemic control and insulin automation	Risk prediction and metabolic prevention	Optimization of diabetes management through data-driven support	T1D focuses on acute glycemic stability; T2D focuses on long-term risk reduction
Main role of AI	Continuous monitoring, insulin dose adjustment, closed-loop support	Longitudinal risk stratification and lifestyle support	Clinical decision support and patient engagement	T1D requires rapid-response systems; T2D requires predictive and preventive models
Acute complications addressed	Hypoglycemia and diabetic ketoacidosis prevention	Acute complications are less frequent	Early detection of metabolic deterioration	T1D has a higher risk of ketoacidosis; T2D rarely presents acute metabolic crises
Chronic complications focus	Secondary focus in pediatric age	Central focus (cardiovascular and metabolic risk)	Prevention of long-term complications	T2D emphasizes cardiovascular risk; T1D emphasizes glycemic variability
Data sources commonly used	Continuous glucose monitors, insulin pumps, wearables	Electronic health records, anthropometric and lifestyle data	Digital health platforms and clinical records	T1D relies on real-time sensor data; T2D relies on longitudinal clinical data
Outcome measures reported	HbA1c reduction, increased time in range, hypoglycemia reduction	Metabolic trends, weight-related outcomes, risk scores	Improvement in clinical decision-making	T1D outcomes are immediate and measurable; T2D outcomes are indirect and heterogeneous
Role of lifestyle interventions	Supportive role	Central role	Education and behavior modification	Lifestyle change is essential in T2D; supportive in T1D
Patient autonomy and self-management	Enhanced through automation and real-time feedback	Enhanced through education and risk awareness	Patient and family empowerment	Autonomy in T1D is technology-driven; in T2D it is behavior-driven
Level of clinical maturity	More advanced and closer to routine use	Less mature and more heterogeneous	Growing evidence base	T1D applications are more clinically integrated than T2D in pediatrics
Main limitations identified	Small cohorts and limited long-term follow-up	Limited pediatric-specific data	Need for prospective pediatric studies	Evidence is stronger for T1D than for pediatric T2D

**Table 4:** Overview of clinical and methodological aspects of artificial intelligence and digital interventions in diabetes care.

Author, Year	Population	AI/Digital Method	Key Clinical Outcomes	Performance Metrics	Main Limitations
(Wong et al., 2018, USA) [1]	Pediatric (T1D)	Data aggregation and visualization platform	Improved clinical workflow and data integration	Mean HbA1c change	No predictive modeling; no direct glycemic outcomes
(Bahal et al., 2024, USA) [2]	Pediatric (T1D)	Narrative synthesis of technological innovation	Improved understanding of contemporary diabetes management	Feasibility metrics	No primary clinical data
(Sarfati et al., 2018, New Zealand) [3]	Mixed (adult T1D/T2D)	Digital behavioral intervention platform	HbA1c and weight improvement	Not reported	Adult-only population; no AI-based prediction
(Curran et al., 2023, Canada) [4]	Pediatric (T1D)	Historical clinical framework	Long-term outcome contextualization	Sensitivity, specificity	No digital or AI component
(Richter et al., 2022, USA) [14]	Mixed (adolescents/adults, T2D)	Mechanistic model with data assimilation	Glycemic state prediction following surgery	Mean HbA1c change	Mixed-age cohort; post-surgical context
(San et al., 2016, Australia) [10]	Pediatric (T1D)	Deep learning (ANN)	Hypoglycemia detection	Not reported	Small sample size; retrospective validation
(Prahalad et al., 2018, USA) [11]	Pediatric (T1D)	Diabetes technologies (CGM, insulin pumps)	Improved HbA1c and patient-reported outcomes	Mean HbA1c change; Hedges’ g	Technology-focused; no standalone AI model
(Fernandez-Luque et al., 2021, Germany) [12]	Pediatric/Mixed	Digital precision medicine platforms	Care optimization and clinical decision support	Not reported	Narrative scope; no quantitative performance metrics
(Nkhoma et al., 2021, Taiwan) [13]	Mixed (T1D/T2D)	Digital education interventions (meta-analysis)	Modest HbA1c and adherence improvement	HbA1c; health literacy scores	Heterogeneous interventions
(Morgado et al., 2025, Brazil) [15]	Pediatric (T1D)	Educational digital technologies	Improved self-management and engagement	Literacy score change	Qualitative outcomes; no AI algorithms
(Wu et al., 2023, China) [16]	Adults (T2D)	AI-assisted education system	Self-management feasibility	AUROC, RMSE	Protocol study; no outcome data
(Naef et al., 2023, Germany) [18]	Pediatric (T1D)	Digital health literacy interventions	Improved health literacy	Mean HbA1c change	Indirect clinical outcomes
(Marcus et al., 2020, Israel) [19]	Mixed (T1D/T2D)	Supervised machine learning	Short-term glucose prediction	Accuracy 100%	Adult-dominant dataset
Calderon Martinez et al., 2024, México) [20]	Pediatric (T1D)	Insulin pump vs. MDI (technology-assisted)	Improved glycemic control	TIR, hypoglycemia rates	No AI-based model
(Spagnolo et al., 2024, Canada) [21]	Pediatric (T1D)	Metabolomic profiling	Identification of DKA biomarkers	R^2^, correlation	Diagnostic focus; no AI classifier
(Daskalaki et al., 2016, Switzerland) [22]	Pediatric (T1D)	Model-free machine learning	Personalized glucose prediction	Sensitivity 78%, specificity 60%	Small cohort
(Stawiski et al., 2018, Poland) [23]	Pediatric (T1D)	ANN (NIRCa)	Insulin resistance estimation	AUROC	Feasibility study
(Ling et al., 2016, Australia) [24]	Pediatric (T1D)	Extreme Learning Machine (ELM)	Hypoglycemia detection	RMSE, correlation	Limited external validation
(Aminian et al., 2020, USA) [25]	Adults (T2D)	Machine learning risk prediction model	Prediction of long-term complications	Mean HbA1c change	Adult-only population
(Daskalaki et al., 2012, Switzerland) [26]	Pediatric (T1D)	Adaptive real-time ML models	Personalized glycemic control	Prediction accuracy	Simulator-based testing
(Esposito et al., 2024, Italy) [27]	Pediatric (T1D)	Telemedicine platforms	Improved access and mixed HbA1c effects	Mean HbA1c change	Variable outcome reporting

## Data Availability

No new data were created or analyzed in this study.

## Supplementary Materials

The following supporting information can be downloaded at: https://www.mdpi.com/article/10.3390/ijms27020802/s1.

## Abbreviations

The following abbreviations are used in this manuscript

ACA	Actor-Critic Algorithm
AI	Artificial Intelligence
AI-HEALS	Artificial Intelligence–Health Evaluation and Learning System
ANNs	Artificial Neural Networks
AOC	Area of Concentration
AP	Artificial Pancreas
AHP	Adhera Health Platform
AR	Autoregressive Model
ARX	Autoregressive with Exogenous Inputs
BDA	Big Data Analytics
BCF	Beta Cell Function
BMF	BetaMe Platform
BMI	Body Mass Index
CGM	Continuous Glucose Monitoring
CI	Confidence Interval
CDSS	Clinical Decision Support Systems
DKA	Diabetic Ketoacidosis
DH	Digital Health
DT	Digital Twin
EHR	Electronic Health Records
FDA	Food and Drug Administration
GB	Gradient Boosting
GPM	Glycemic Prediction Model
GC	Glycemic Control
HbA1c	Hemoglobin A1c
IPT	Insulin Pump Therapy
LSTM	Long Short-Term Memory
MAE	Mean Absolute Error
MDL	Multimodal Deep Learning
ML	Machine Learning
NLP	Natural Language Processing
NIRCa	Non-Invasive Recording Calculator
PAI	Predictive Artificial Intelligence
PRISMA	Preferred Reporting Items for Systematic Reviews and Meta-Analyses
RF	Random Forest
ROC	Receiver Operating Characteristic
RMSE	Root Mean Square Error
RNNs	Recurrent Neural Networks
SD	Standard Deviation
SVM	Support Vector Machines
T1DM	Type 1 Diabetes Mellitus
T2DM	Type 2 Diabetes Mellitus
TIR	Time in Range
USA	United States of America
UK	United Kingdom
PHR	Personal Health Record
