# Supplementary material for: Artificial Intelligence-Driven Transformation of Pediatric Diabetes Care: A Systematic Review and Epistemic Meta-Analysis of Diagnostic, Therapeutic, and Self-Management Applications
Source: Int J Mol Sci. 2026 Jan 13;27(2):802. doi: 10.3390/ijms27020802 (PMC12841495; doi:10.3390/ijms27020802)
Supplement: Supplementary file 1 [file ijms-27-00802-s001.zip › Table S2.pdf]

Author, Year	Base data	Input	Output
(Wong et al., 2018, USA) (1)	Diabetes device data (e.g., CGM, insulin pumps)	Integration and visualization through Tidepool platform	Patient monitoring and care coordination
(Bahal et al., 2024, USA) (2)	Clinical use of CGM systems and insulin pumps in children	Technological innovation in real-time monitoring and insulin delivery	Glycemic control and personalized treatment
(Sarfati et al., 2018, New Zealand) (3)	Self-reported data via web/mobile platform	Behavioral change support and education through digital tools	Self-management and lifestyle intervention
(Laron et al., 1989, Israel) (17)	Historical and epidemiological data	Literature review and global trends analysis	Epidemiological insight on T1D in children
(Curran et al., 2023, Canada) (4)	Global health data and prevalence reports (e.g., IDF)	Analysis of diabetes incidence and access to treatment	Health policy and access recommendations
(Richter et al., 2022, USA) (14)	Clinical data from adolescents undergoing bariatric surgery	Mechanistic models and machine learning assimilation	Glycemic state prediction post-surgery
(San et al., 2016, Australia) (10)	ECG signals (HR, QTc) from 15 children with T1D	Deep learning model (DBN) to detect hypoglycemia	Hypoglycemia prediction and diagnostics
(Prahalad et al., 2018, USA) (11)	Review of clinical outcomes and patient-reported measures	Evaluation of diabetes technologies and digital tools	Improved care delivery and psychosocial outcomes
(Fernandez-Luque et al., 2021, Germany) (12)	Literature and case studies on pediatric endocrine disorders	Digital health integration for precision medicine	Personalized treatment and patient engagement
(Nkhoma et al., 2021, Taiwan) (13)	Meta-analysis of digital education interventions for T1D and T2D	Systematic review of self-management education platforms	Education for self-management and improved HbA1c
(Morgado et al., 2025, Brazil) (15)	Content developed and validated with families and experts	Development of printed/digital educational booklet	Family education for pediatric T1D care
(Wu et al., 2023, China) (16)	Protocol design for AI-powered educational system (AI-HEALS)	Development of AI-guided self-management support	Personalized education and behavior reinforcement
(Naef et al., 2023, Germany) (18)	Systematic review of digital interventions for adolescents with T1D	Evaluation of digital tools to improve health literacy	Improved health literacy and self-care behavior
(Marcus et al., 2020, Israel) (19)	Clinical glucose data from patients at Tel Aviv Medical Center	Machine learning model to predict glucose levels	Blood glucose prediction
Calderon Martinez et al., 2024, México) (20)	Pediatric studies comparing insulin pump vs. injections	Systematic review and meta-analysis	Treatment efficacy comparison
(Spagnolo et al., 2024, Canada) (21)	Metabolomic profiles of pediatric DKA patients	NMR and mass spectrometry analyzed with ML tools	Biomarker discovery and diagnostic support

(Daskalaki et al., 2016, Switzerland) (22)	Simulated T1D patient data using FDA-approved simulator	Reinforcement learning (Actor-Critic) to optimize insulin delivery	Personalized insulin dosing optimization
(Stawiski et al., 2018, Poland) (23)	Clamp test data from 315 children with T1D	ANN and MARSplines to estimate insulin resistance	Insulin resistance estimation
(Ling et al., 2016, Australia) (24)	ECG data from 16 children with T1D	Extreme Learning Machine model for classification	Hypoglycemia monitoring
(Aminian et al., 2020, USA) (25)	Clinical data of >13,000 patients with T2D with/without surgery	Random Forest ML to predict complications	10-year risk prediction of complications
(Daskalaki et al., 2012, Switzerland) (26)	Real-time glucose and insulin data from T1D patients	Adaptive ANN and AR/ARX models	Glycemic prediction and hypoglycemia detection
(Esposito et al., 2024, Italy) (27)	Review of studies on pediatric T1D during COVID-19	Narrative review of telemedicine applications	Recommendations for digital management of pediatric diabetes
