# Supplementary material for: Artificial Intelligence-Driven Transformation of Pediatric Diabetes Care: A Systematic Review and Epistemic Meta-Analysis of Diagnostic, Therapeutic, and Self-Management Applications
Source: Int J Mol Sci. 2026 Jan 13;27(2):802. doi: 10.3390/ijms27020802 (PMC12841495; doi:10.3390/ijms27020802)
Supplement: Supplementary file 1 [file ijms-27-00802-s001.zip › Table S1.pdf]

Title, Year, Country	Population	Intervention	Comparassion	Results	Basal Risk
(Wong et al., 2018, USA) (1)	15 health care providers and pediatric patients with type 1 diabetes	Use of the Tidepool software platform to integrate data from multiple diabetes devices over 6 months	Usual practice using proprietary software from device manufacturers	Increased provider reference to data (from 2.8 to 6.1 times per visit, p=0.0002); increased percentage of children viewing data during visits (from 61% to 94%, p=0.015); no increase in visit time or administrative burden	Patient age with diabetes mellitus: P-0.47. Patient gender (female) P- 0.64. Patients using CGM: P: 0.08. Blood glucose checks per day: P-017. HA1c%: P: 0.66
(Bahal et al., 2024, USA) (2)	Children and adolescents with Type 1 Diabetes, comprehensive literature search.	CGM, insulin pumps, artificial pancreas, ultrarapid-acting insulins, adjunctive pharmacotherapies (SGLT2i, GLP-1 RA), immunotherapy, stem cells, digital health tools, AI, genetic precision medicine	Terapias tradicionales de control de hiperglusemia vs avanes tecnológicos.	Improved glycemic control, increased time-in-range, reduced HbA1c and hypoglycemia, enhanced quality of life; promising future therapeutic targets	Not mention
(Sarfati et al., 2018, New Zealand) (3)	Adults aged 18–75 with Type 2 Diabetes Mellitus or pre-diabetes (n=430)	12-month digital self-management programme including health coaching, goal tracking, peer support, and educational content (BetaMe)	Usual care (primary care consultations and education)	Primary outcomes: change in HbA1c and weight at 12 months; also improvements in waist circumference, BP, self-care behaviors, and quality of life; pilot showed ↓HbA1c, ↓weight	Not mention
(Laron et al., 1989, Israel) (17)	Children and adolescents with Type 1 Diabetes	Historical review of epidemiology, genetic-environmental etiology, diagnostic advances, and therapeutic strategies (e.g., insulin therapy, education, monitoring, prevention efforts)	Early 20th century diabetes care	Improved metabolic control, reduced complications, advances in screening, recognition of psychosocial impacts; therapy access still unequal globally	Not mention
(Curran et al., 2023, Canada) (4)	Children and young adults (3–26 years) with T1DM or T2DM in Bangladesh	AI-based screening for diabetic retinopathy (Cybersight AI); interpretation of macula-centered fundus images	Reference standard: DR-certified optometrist using ICDR grading	AI detected any DR in 19.4% and referable DR in 2.35%; high sensitivity (75.5–84.2%) and specificity (91.8–98.9%); MCC = 63.4–66.4	Gender: Male: P-0.62 (0.23-1.63). Female: 0.328. Severity of DR:No DR: P-Reference, Mild: P-0.05 (0.01-0.17), Moderate: P-0.02. Age: P-0.65 (0.54-0.76) years. Diabetes duration: P 0.82 (0.75-0.91) years

(Richter et al., 2022, USA) (14)	Adolescents (10–21 years) with severe obesity undergoing bariatric surgery (n=176)	Data assimilation combined with mechanistic modeling (insulin sensitivity and secretion) to predict glycemic status post-surgery	Models using only clinical/biochemical data (e.g., HOMA-IR, OGTT)	Best model achieved AUROC had impaired glucose metabolism at 12 months; model performance improved over HOMA-IR	Pre-operative IGM status P < 0.0001. Race: Asian: P-0.1000, Black: P. 0.1390, Native American: P- 0.3180, Other: P - 0.2110, Pacific Islander: P- 1.0000, White: P- 0.1890, Unknown: P- 0.5890. Ethnicity: Hispanic: P- 0.3220, Not Hispanic: 0.9760, Declined: P- 1.0000, Unknown: P- 0.1060. Surgery Type: RYGB: P- 1.0000, AGB: P- 0.0370, VSG: P- 0.0600, Other: P- 1.0000. ICD codes: Abnormal Glucose: P- 0.0030, DM2: P- 0.0130, Dyslipidemias: P- 0.4850, GERD: P.1.0000, HAS: P-0.0130, Liver Disease: P- 0.6780, MS: P- 0.8220, Thyroid disease: 0.2930. Baseline HbA1c (%) P < 0.0001 Baseline HOMA-IR P-1.987 × 10–3. Other Labs: Total Cholesterol P- 0.6057 Triglycerides P- 0.4017 ALT P- 0.3676 AST P- 0.8286 TSH P- 0.4416 Free T4 P- 0.2113 Total T4 P- 0.3143
(San et al., 2016, Australia) (10)	15 children with Type 1 Diabetes	Deep Belief Network (DBN) trained with heart rate (HR) and corrected QT (QTc) from ECG signals to detect nocturnal hypoglycemia	BBNN, WNN, FFNN, MR models	DBN showed best performance: Sensitivity 80%, Specificity 50%, outperforming other models in detection of hypoglycemic episodes	Studies on the naturaloccurrence of hypoglycemia with an increase in heart rate,(1.033±0.242 vs. 1.082±0.298, P<0.06) and corrected QT intervals, (1.031±0.086 vs. 1.060±0.084, P < 0.01)

(Prahalad et al., 2018, USA) (11)	Children and adolescents with Type 1 Diabetes	Use of diabetes technology: CGM, insulin pumps, hybrid closed-loop systems, telemedicine, digital health apps, big data	Traditional SMBG and MDI therapy	Improved glycemic control (HbA1c), fewer hypoglycemic and DKA events, enhanced psychosocial outcomes and patient satisfaction	Retinopathy % 5.6 (4.4–7.0) Nephropathy % 5.8 (4.6–7.4) Neuropathy % 8.5 (7.1–10.2) Hypertension% 10.1 (8.6–11.9) Hyperlipidaemia % 14
(Fernandez-Luque et al., 2021, Germany) (12)	Digital Health for Supporting Precision Medicine in Pediatric Endocrine Disorders: Opportunities for Improved Patient Care	Integration of digital health technologies (eHealth, telemedicine, AI, genomic tools, chatbots, apps, AR) into precision medicine	Traditional "one-size-fits-all" healthcare approaches	Potential to improve diagnostic precision, patient education, adherence (e.g. GH therapy), glycemic control, and outcomes; examples include AI for growth tracking, glucose monitoring, digital communication	Not mention
(Nkhoma et al., 2021, Taiwan) (13)	6,861 participants (mean age: 51.6, range: 13–70), including T1DM and T2DM	Digital DSMES (mobile apps, web portals, social media)	Usual care or traditional DSMES	HbA1c ↓ at 6 months (T2DM: -0.49%, T1DM: -0.42%); 12 months (T2DM: -0.59%, T1DM: -0.03%). Knowledge ↑ at 3 months (g=1.003); HrQoL effect small and nonsignificant	6 months to 12 months of follow-up P – value: HbA1c 4 overall P 0.00, HbA1c Type 1 P 0.20, HbA1c Type 2 P 0.00, HbA1c Mobile P 0.000, HbA1c Social Media P 0.000, HrQoL P 0.106, HbA1c overall P 0.000, DM 4 Knowledge P 0.036
(Morgado et al., 2025, Brazil) (15)	Children with T1D and their families (studies included children aged 2–12 and caregivers)	Digital and non-digital educational technologies: videos, games, apps, simulators, booklets, workshops, toys	Conventional health education or no specified comparator	ETs improved knowledge, self-care skills, interaction with professionals; evidence for long-term effectiveness and adherence is still limited	Not mention
(Wu et al., 2023, China) (16)	Adults (18–75 years) with Type 2 Diabetes in community health centers (Beijing)	AI-HEALS via WeChat: KBQA chatbot, lifestyle + glucose tracking, reminders, automated education messages	Standard diabetes primary care (SDPC)	Expected: ↓ HbA1c; ↑ self-management, health literacy, medication adherence; cost-effectiveness evaluated at 12 and 18 months	Not mention
(Naef et al., 2023, Germany) (18)	Adolescents (13–19 years) with Type 1 Diabetes (22 included studies)	Distal digital health technologies (social media, mHealth, telehealth, apps, games, messaging systems)	Standard care or non-digital interventions	Improvement in communication, knowledge, engagement, and self-management; "communication and interaction" was the most reported health literacy dimension (25.6%)	Not mention
(Marcus et al., 2020, Israel) (19)	11 adults with T1DM (aged 18–39) using CGM	Supervised machine learning algorithm (kernel ridge regression variants) for predicting 30-min glucose values	Naïve algorithms (mean and last value predictions); prior ML models	RMSE: 20.5 mg/dL; AME: 15.4 mg/dL; Accuracy: 93.4%; True-positive hypoglycemia prediction rate: 64%; False-positive: <4%;	Not mention

				superior to baseline models	
Calderon Martinez et al., 2024, México) (20)	Children and adolescents with Type 1 Diabetes (n=3,512 from 23 studies)	Continuous Subcutaneous Insulin Infusion (CSII) via insulin pump	Multiple Daily Injections (MDI)	Qualitative findings: 61% of studies showed improved HbA1c with CSII. Meta-analysis: No statistically significant difference in HbA1c (mean diff 0.22; 95% CI: -0.038 to 0.48); high heterogeneity	The test for heterogeneity yielded a Q value of 61.29 with 13 degrees of freedom, demonstrating significant heterogeneity ($p<0.0001$)
(Spagnolo et al., 2024, Canada) (21)	34 pediatric T1D patients: 17 with severe DKA, 17 insulin-controlled	Metabolomics profiling (NMR and DI-LC/MS/MS) to distinguish metabolic profiles	Insulin-controlled T1D patients	65 metabolites differed significantly; key metabolites: ketones, acylcarnitines, phosphatidylcholines; metabolite panels associated with pH, bicarbonate, glucose, HbA1c, and GCS; pathway enrichment: ketone synthesis, butanoate, arginine, tyrosine, proline metabolism	Mean age: P - 0.96, Male: female ratio P. 1.00, BMI z-SCORE p- 0.70. GCS P <0.001, HbA1c % P <0.001, Glucose (mmoL/L) P <0.001
(Daskalaki et al., 2016, Switzerland) (22)	Children, adolescents, and adults with T1D (simulated patients, n=128 total across cohorts)	Actor-Critic (AC) reinforcement learning algorithm for glucose control via insulin pump using CGM data	Open-loop standard insulin therapy with fixed BR and IC ratios	AC improved time-in-range (up to 95.66%), reduced mild/severe hypoglycemia (e.g., <0.3%), and adapted to SI variation and meal uncertainty	Manual and TE-based initialisation in hypoglycaemia prevention were statistically different in both adults and children (p values<0.05)
(Stawiski et al., 2018, Poland) (23)	Children and adolescents with Type 1 Diabetes (n=315; age 7.6–19.7 years)	ANN model for estimating insulin resistance (GDR) using clinical parameters (e.g., BMI, insulin dose, HbA1c)	Traditional estimation models (e.g., Dabelea model using waist, TG, HbA1c)	ANN achieved best accuracy (median error 0.6%, R^2=0.66); within ±20% error in 75% of cases; outperformed MARSplines and reference models	Predictions of ANN, showed significantly lower error than ARSplines (P < .0001) and better fit regardless of patient age. Sex: Male. P - .1129. Tanner Stege 1: P- .4056, Prehypertensive state: P- .8632, Hypertension: P- .8405
(Ling et al., 2016, Australia) (24)	16 children with Type 1 Diabetes (mean age 14.6 ± 1.5 years)	Non-invasive monitoring system using ECG parameters (HR, QTc, ΔHR, ΔQTc) and Extreme Learning Machine (ELM) neural network	Other models: PSO-NN, MR-FIS, FIS, LMR	ELM-NN outperformed others: Sensitivity 78%, Specificity 60%, fastest training time (3 min); reliable detection of nocturnal hypoglycemia	Results indicated that the degree of QTclengthening during clamped hypoglycemia (583ms) was greater vs euglycemic control (492 ms) P < 0.001

(Aminian et al., 2020, USA) (25)	13,722 adults with T2DM and obesity (2,287 surgical; 11,435 nonsurgical)	IDC Risk Score: ML and regression models using EHR to predict complications with/without metabolic surgery	RECODe risk models and standard care without ML	ML models had AUC 0.66–0.81 across outcomes (mortality, coronary events, HF, nephropathy); superior to RECODe in IPA, calibration, AUC	Not mention
(Daskalaki et al., 2012, Switzerland) (26)	Simulated data from 30 patients (10 adults, 10 adolescents, 10 children) using the UVA/PADOVA T1D simulator	Adaptive autoregressive (AR), ARX (with insulin), and artificial neural network (ANN) models for glucose prediction	AR and ARX vs ANN performance	ANN outperformed AR/ARX in RMSE, time lag, and hypoglycemia prediction: sensitivity 96% and specificity 99% (PH=30min); RMSE as low as 2.8 mg/dL	Not mention
(Esposito et al., 2024, Italy) (27)	Children and adolescents with Type 1 Diabetes	Telemedicine (video consults, data uploads, asynchronous/synchronous TM, mHealth apps, remote monitoring)	Traditional in-person consultations	Mixed results: ↑ consultation frequency, ↑ satisfaction, ↓ costs, ↓ HbA1c in some studies; inconsistent HbA1c effects across time points (effective at 6 months, not at 3 or 12 months); improved access and emotional well-being	Insulin pump users vs multiple daily injections (92.4% vs. 82.5%; P = 0.023). Satisfaction levels higher for families living farther healthcare providers (97.7% vs. 89.1%; P = 0.017 Frequency of diabetes visits from 3.8 ± 1.7 to 4.3 ± 2.2 per year (P < 0.001) Reduction in HbA1c levels by 0.22 (P < 0.001) vs Subgroup analysis TM was not effective at 3 months (P = 0.07) or at 12 months (P = 0.85) at the 6-month mark (P = 0.01)
