# Supplementary figures and images for: Artificial Intelligence-Driven Transformation of Pediatric Diabetes Care: A Systematic Review and Epistemic Meta-Analysis of Diagnostic, Therapeutic, and Self-Management Applications
Source: Int J Mol Sci. 2026 Jan 13;27(2):802. doi: 10.3390/ijms27020802 (PMC12841495; doi:10.3390/ijms27020802)

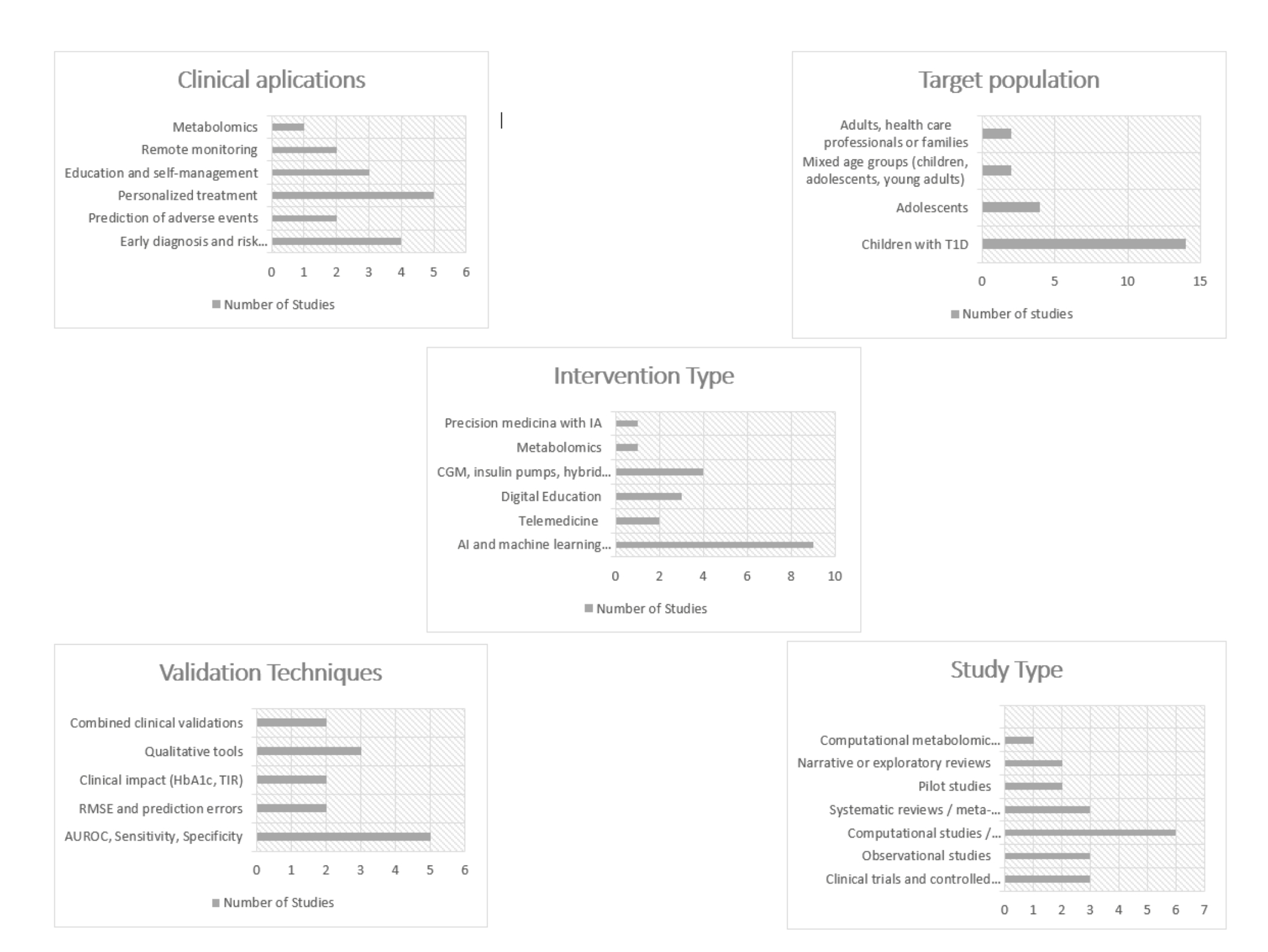

Supplement: Supplementary file 1 [file ijms-27-00802-s001.zip › Figure S1.tiff]
